# MicroRNA-146a regulates ICOS–ICOSL signalling to limit accumulation of T follicular helper cells and germinal centres

**DOI:** 10.1038/ncomms7436

**Published:** 2015-03-06

**Authors:** Alvin Pratama, Monika Srivastava, Naomi J. Williams, Ilenia Papa, Sau K. Lee, Xuyen T. Dinh, Andreas Hutloff, Margaret A. Jordan, Jimmy L. Zhao, Rafael Casellas, Vicki Athanasopoulos, Carola G. Vinuesa

**Affiliations:** 1 Department of Pathogens and Immunity, John Curtin School of Medical Research, Australian National University, Building 131, Garran Road, Canberra, Australian Capital Territory 0200, Australia; 2 Comparative Genomics Centre, James Cook University, Townsville, Queensland 4811, Australia; 3 Chronic Immune Reactions Group, German Rheumatism Research Centre Berlin (DRFZ), a Leibniz Institute, 10117 Berlin, Germany; 4 Division of Biology and Biological Engineering, California Institute of Technology, Pasadena, California 91125, USA; 5 Genomics and Immunity Section, National Institute of Arthritis and Musculoskeletal and Skin Diseases, National Institutes of Health, Bethesda, Maryland 20892, USA

## Abstract

Tight control of T follicular helper (Tfh) cells is required for optimal maturation of the germinal centre (GC) response. The molecular mechanisms controlling Tfh-cell differentiation remain incompletely understood. Here we show that microRNA-146a (miR-146a) is highly expressed in Tfh cells and peak miR-146a expression marks the decline of the Tfh response after immunization. Loss of miR-146a causes cell-intrinsic accumulation of Tfh and GC B cells. MiR-146a represses several Tfh-cell-expressed messenger RNAs, and of these, ICOS is the most strongly cell autonomously upregulated target in miR-146a-deficient T cells. In addition, miR-146a deficiency leads to increased ICOSL expression on GC B cells and antigen-presenting cells. Partial blockade of ICOS signalling, either by injections of low dose of ICOSL blocking antibody or by halving the gene dose of *Icos* in miR-146a-deficient T cells, prevents the Tfh and GC B-cell accumulation. Collectively, miR-146a emerges as a post-transcriptional brake to limit Tfh cells and GC responses.

## Abstract (web summary)

Maturation of antibody-producing B cells in germinal centers is orchestrated by T follicular helper cells. Here Pratama *et al*. show that miR-146a negatively regulates T follicular helper cells by targeting ICOS-ICOS ligand signaling in germinal centers.

## Graphical abstract

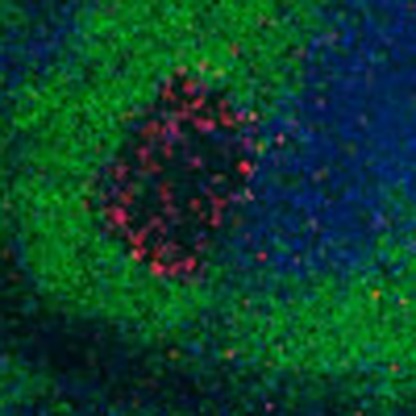

T follicular helper (Tfh) cells provide essential survival and selection signals to germinal centre (GC) B cells that are important for long-lived protective antibody responses^1^,^2^. Increasing evidence suggests that restricting Tfh-cell numbers in GCs is crucial for optimal GC B-cell selection^3^,^4^,^5^. B cells expressing the highest affinity receptors after somatic hypermutation can capture more antigens and therefore have a competitive advantage in establishing sustained interactions and eliciting survival signals from Tfh cells^5^. Studies of autoimmune mouse models^6^,^7^,^8^,^9^ and human patients^10^,^11^,^12^,^13^,^14^ suggest that excessive Tfh cells may contribute to the pathogenesis of antibody-mediated autoimmune diseases, potentially by allowing survival and differentiation of self-reactive B cells. While multiple signals are now recognized to be important for Tfh-cell formation and migration^3^, relatively little is known about the mechanisms that limit Tfh-cell numbers to achieve optimal selection of high affinity B-cell clones. Cell-extrinsic mechanisms such as the actions of T follicular regulatory (Tfr)^15^,^16^,^17^ and follicular CD8^+^ T cells^18^ have been reported, but to date, only Roquin is shown to act in a T cell-autonomous manner to prevent spontaneous accumulation of Tfh cells^19^.

MicroRNA-146a (miR-146a) has recently emerged as a key post-transcriptional regulator in hematopoietic cells. MiR-146a represses TRAF6 and IRAK1 in myeloid cells^20^ and T cells^21^ to control their proliferation and NF-κB activation in response to Toll-like receptor and TCR signalling, respectively. Deficiency of miR-146a leads to excessive production of IL-6 and TNF, myeloproliferation, chronic inflammation and a decline in the number and quality of hematopoietic stem cells^20^,^22^,^23^. In the absence of miR-146a, regulatory T (Treg) cells also lose their suppressive ability due to STAT1 overexpression driving increased IFN-γ secretion^24^. Not surprisingly, dysregulated expression of miR-146a has also been found to correlate with increased incidence of autoimmune diseases, such as lupus^25^,^26^,^27^,^28^ and rheumatoid arthritis^29^,^30^,^31^,^32^.

Here we show that miR-146a profoundly represses Tfh-cell numbers: the absence of this miRNA leads to spontaneous Tfh-cell accumulation that precedes myeloid cell dysregulation and is not a consequence of Treg-cell functional deficiency. This is achieved by directly repressing multiple messenger RNAs (mRNAs) targets, most prominently *Icos*.

## Results

### MiR-146a is highly expressed in Tfh cells

**Figure 1:**
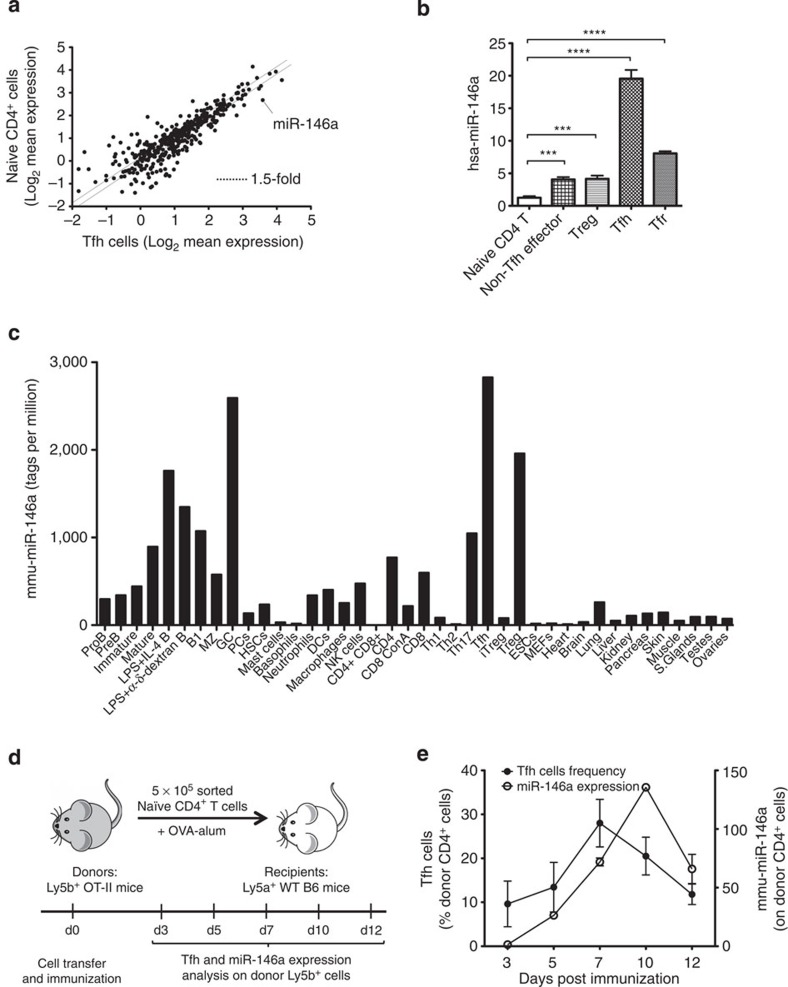
MiR-146a is highly expressed in Tfh cells. (**a**) Microarray analysis of miRNA expression in naïve CD4^+^ T and Tfh cells. The scatter plot shows the log_2_ of averaged (*n*=3) background-subtracted expression of all Agilent Human miRNA Microarray (V1) Kit 8 × 15K probes by Tfh cells on the *x*-axis compared with naïve CD4^+^ T cells on the *y*-axis. Each dot represents one miRNA probe. The dotted lines are drawn at 1.5-fold difference in expression. (**b**) Expression of miR-146a, normalized to U6, measured by real-time PCR from human tonsillar T-cell subsets. Cell sorting strategy is described in the Methods. The heights of the bars represent the mean, and the error bars represent the s.d. of three technical replicates. Data are representative of three independent experiments. Statistical significance was determined using unpaired Student’s *t*-test. ***P<0.001, ****P<0.0001. (**c**) Bar graphs showing the expression of miR-146a in mouse immune cell subsets and non-hematopoietic tissues as described in Kuchen *et al*.^33^. In brief, small RNAs from various mouse tissues were ligated to adapters using Illumina’s protocol. These were then reverse transcribed, amplified by PCR and sequenced on a Genome Analyzer (Illumina). (**d**,**e**) Kinetics of the Tfh-cell response (filled circles) and miR-146a expression (open circles) from transferred Ly5b^+^ OT-II cells analyzed at different time points according to the strategy shown in **d**. Three recipient mice were killed at each time point. Percentage of Tfh cells among donor Ly5b^+^ cells was measured using flow cytometry staining of CD4^+^ CXCR5^high^ PD-1^high^ Foxp3^−^ T cells. MiR-146a expression was measured by real-time PCR of total Ly5b^+^ CD4^+^ T cells and was normalized to U6 expression. Filled and open circles in **e** represent the mean, and error bars represent the s.d. of biological replicates (for Tfh-cell percentage) or technical replicates (for miR-146a expression).

To identify miRNAs that might regulate Tfh-cell development and/or function, we performed a genome-wide expression analysis to identify miRNAs that are highly expressed in human Tfh cells. Compared with naïve (CD44^low^ CD25^−^) CD4^+^ T cells, miR-146a was the most differentially expressed miRNA (8.2-fold) in Tfh cells (Fig. 1a) among those that are highly expressed in T cells. Real-time PCRs on T-cell subsets from human tonsils revealed that Tfh cells expressed the highest amounts of miR-146a compared with all other effector (CD44^high^) subsets (Fig. 1b). Consistent with these results, a deep-sequencing survey of the miRNome in mouse tissues showed the highest levels of miR-146a in Tfh and GC B cells (Fig. 1c and ref. 33). Lower amounts of miR-146a were also present in activated B cells and thymic-derived Tregs, while non-haematopoietic tissues displayed little or no miR-146a expression (Fig. 1c).

### MiR-146a expression peaks at the late stage of GC responses

To study the kinetics of miR-146a expression during a T cell-dependent immune response, we adoptively transferred 5 × 10^5^ naïve CD4^+^ T cells from Ly5b^+^ OT-II TCR-transgenic mice into Ly5a^+^ wild-type C57BL/6 recipient mice, which were then immunized with ovalbumin (OVA) precipitated in alum and killed at different time points (Fig. 1d). The proportion of Tfh cells among donor cells peaked at day 7 post immunization (Fig. 1e) and started to contract by day 10, coinciding with the peak of miR-146a expression in transferred cells (Fig. 1e). This suggests a possible role for miR-146a in downsizing the Tfh-cell population.

### Cell-autonomous control of Tfh cells by miR-146a

**Figure 2:**
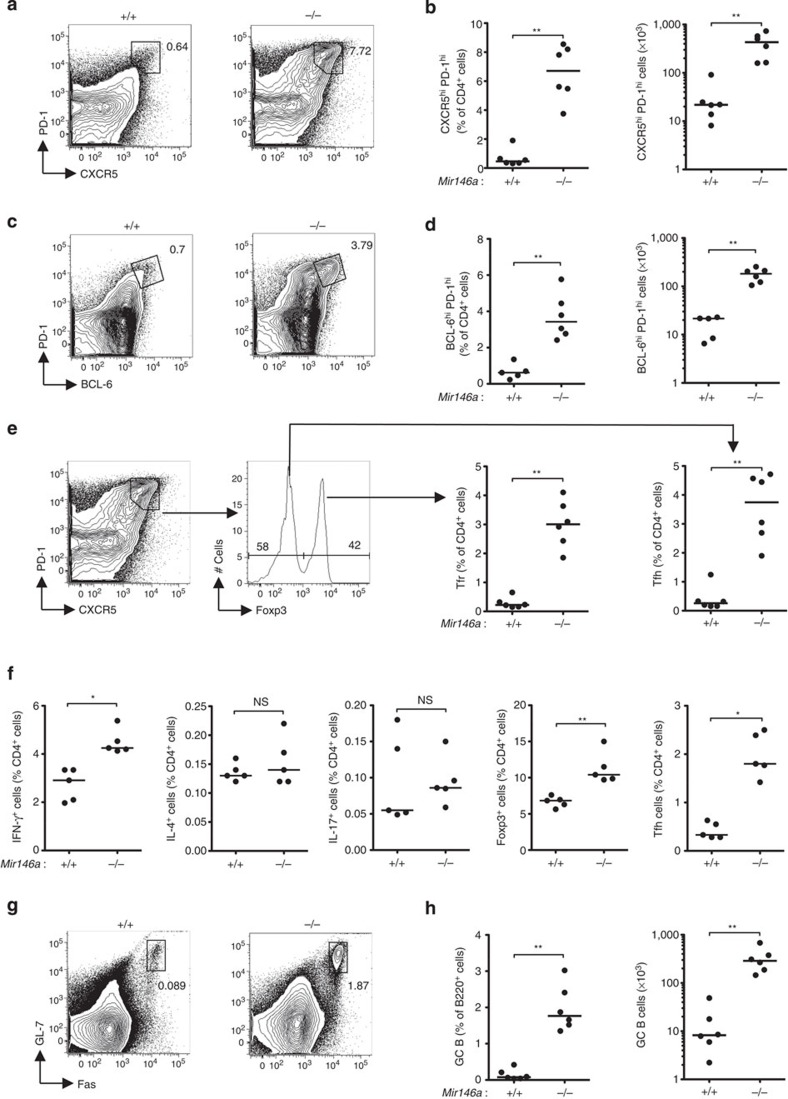
Loss of miR-146a causes spontaneous accumulation of Tfh and GC B cells. (**a**–**d**) Flow cytometric contour plots and dot plots showing percentages and absolute cell number of follicular T cells gated as CXCR5^high^ PD-1^high^ cells (**a**,**b**) or BCL-6^high^ PD-1^high^ cells (**c**,**d**). (**e**) Gating strategy for Tfh (CXCR5^high^ PD-1^high^ Foxp3^−^) and Tfr (CXCR5^high^ PD-1^high^ Foxp3^+^) cells and dot plots showing their percentages. (**f**) Percentages of Th1 (IFN-γ^+^), Th2 (IL-4^+^), Th17 (IL-17^+^), Treg (Foxp3^+^) and Tfh (CXCR5^high^ PD-1^high^ Foxp3^−^) cells among total CD4^+^ cells. (**g**,**h**) Flow cytometric contour plots and dot plots showing percentages and absolute cell numbers of GC B cells. Contour plots shown in **a**,**c**,**e** are gated on CD4^+^ cells, those in **g** are gated on B220^+^ cells. Unimmunized miR-146a-deficient (−/−) and -sufficient (+/+) mice were analyzed. Each symbol represents one mouse and the horizontal bars represent the median values. Data are representative of at least three independent experiments. Statistical significance was determined using Mann–Whitney *U*-test. NS, not significant, *P<0.05, **P<0.01.

To test whether miR-146a plays a role in the control of Tfh-cell numbers, we studied Tfh-cell development and function in 12- to 16-week-old mice lacking miR-146a. Analysis of total follicular T cells, gated as CXCR5^high^ PD-1^high^ (Fig. 2a,b) or BCL-6^high^ PD-1^high^ (Fig. 2c,d) cells, showed they were expanded in both percentage and total number in unimmunized *Mir146a*^−*/*−^ mice compared with wild-type littermates. This expansion was not selective to either Tfh (CXCR5^high^ PD-1^high^ Foxp3^−^) or Tfr (CXCR5^high^ PD-1^high^ Foxp3^+^) cells as both subsets increased in parallel (Fig. 2e). This effect of miR-146a deficiency seemed to affect Tfh cells more severely than the other effector CD4^+^ T cell subsets: compared with the 5-fold increase in Tfh cells, no statistically significant increases were observed in either Th2 (IL-4^+^) cells or Th17 (IL-17^+^) cells, while Th1 (IFN-γ^+^) cells were increased by 1.4-fold and Tregs (Foxp3^+^), as previously reported^24^, were expanded by 1.5-fold (Fig. 2f). Total GC B cells were also increased by about 20-fold in *Mir146a*^−*/*−^ mice compared with wild-type littermates (Fig. 2g,h). The spontaneous accumulation of Tfh and GC B cells became more severe with age (Supplementary Fig. 1).

**Figure 3:**
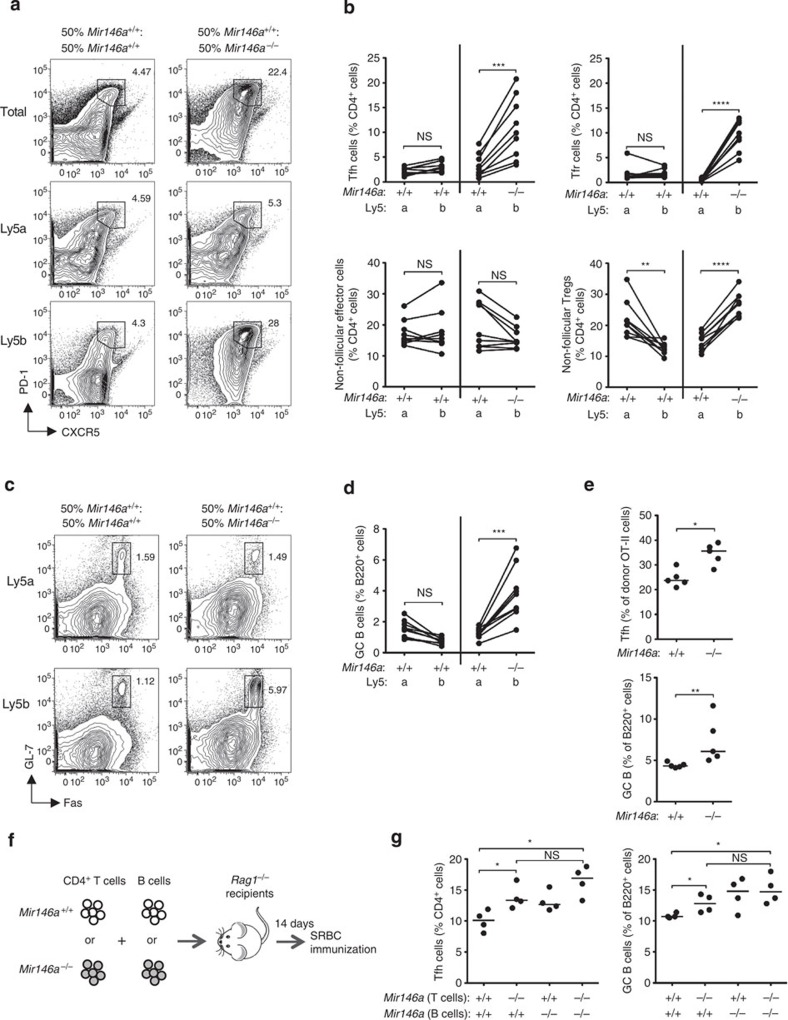
Cell-autonomous accumulation of miR-146a-deficient Tfh cells. (**a**) Flow cytometric contour plots showing the gating strategy for follicular T cells in 50:50 mixed bone marrow chimeras gated on all CD4^+^ (top), Ly5a^+^ CD4^+^ (middle) and Ly5b^+^ CD4^+^ (bottom) cells. (**b**) Percentages of Tfh, Tfr, non-follicular effector and non-follicular Tregs in the mixed chimeras. (**c**,**d**) Flow cytometric contour plots and dot plot showing the percentages of GC B cells in the mixed chimeras. Contour plots in **c** are gated on Ly5a^+^ B220^+^ and Ly5b^+^ B220^+^ cells as indicated. Connecting lines between percentages of Ly5a^+^ and Ly5b^+^ in **b** and **d** indicate that they are from the same chimeric mouse. Chimeras were analyzed 16 weeks post reconstitution. (**e**) Percentages of OVA-specific (Vβ5^+^) Tfh cells and total GC B cells in *Cd28*^−*/*−^ recipient mice 7 days following adoptive transfer. Data are representative of three independent experiments. (**f**,**g**) Adoptive transfer of purified CD4^+^ T cells and B cells from *Mir146a*^*+/+*^ or *Mir146a*^−*/*−^ donor mice into *Rag1*^−*/*−^ recipients according to the strategy shown in **f**. The recipient mice were immunized with sheep red blood cells 14 days after transfer and they were killed 7 days post immunization. (**g**) Percentages of Tfh and GC B cells in *Rag1*^−*/*−^ recipient mice following adoptive transfer and SRBC immunization. Each symbol represents one mouse and the horizontal bars represent the median values. Statistical significance was determined using a paired Student’s *t*-test (**b**,**d**) or Mann–Whitney *U*-test (**e**,**g**). NS, not significant, *P<0.05, **P<0.01, ***P<0.001, ****P<0.0001.

To test whether the accumulation of Tfh cells in *Mir146a*^−*/*−^ mice was due to T-cell intrinsic factors, we set up mixed bone marrow chimeras by transferring equal amounts of either Ly5a^+^.*Mir146a*^*+/+*^: Ly5b^+^.*Mir146a*^*+/+*^ (WT:WT) or Ly5a^+^.*Mir146a*^*+/+*^: Ly5b^+^.*Mir146a*^−*/*−^ (WT:KO) bone marrow cells into sub-lethally irradiated *Rag1*^−*/*−^ recipients. Four months after reconstitution, the spleens were harvested and the percentage of total CD4^+^ T cells, effector cells, Tfh cells, total B220^+^ cells and GC B cells derived from the Ly5a^+^ or Ly5b^+^ donor marrow in each mouse was determined (Fig. 3a–d). There was a higher percentage of total follicular T cells in the WT:KO chimeras compared with the WT:WT chimeras (Fig. 3a). This was due to cell-autonomous expansions of Tfh and Tfr cells derived from Ly5b^+^.*Mir146a*^−*/*−^ marrow (Fig. 3b). In each mouse, there was a two to fourfold increase in Ly5b^+^ Tfh cells compared with Ly5a^+^ Tfh cells, whereas this was not observed in the control WT:WT chimeras, in which the percentages of Ly5a^+^ and Ly5b^+^ Tfh cells were comparable (Fig. 3b). Importantly, the percentages of non-follicular effector CD4^+^ cells were comparable between Ly5a^+^ and Ly5b^+^ cells in both WT:WT and WT:KO mixed chimeras. Consistent with previous reports^24^, there was a cell-autonomous expansion of non-follicular Tregs derived from Ly5b^+^.*Mir146a*^−*/*−^ marrow in the WT:KO chimeras (Fig. 3b).

We also observed a statistically significant increase in the percentage of GC B cells derived from Ly5b^+^.*Mir146a*^−*/*−^ marrow compared with those derived from Ly5a^+^.*Mir146a*^*+/+*^ bone marrow (Fig. 3c,d), suggesting that miR-146a also acts cell autonomously in GC B cells. Intriguingly, despite the significant increase of total follicular T cells in the WT:KO chimeras (Fig. 3a), we only observed expansion of the Ly5b^+^.*Mir146a*^−*/*−^ GC B cells, while the percentage of the neighbouring Ly5a^+^.*Mir146a*^*+/+*^ GC B cells was comparable to that in the WT:WT chimeras (Fig. 3d). This could indicate that GC expansion requires the concerted actions of miR-146a in T cells and B cells, perhaps through the regulation of a receptor–ligand pair in each cell type. Collectively, these results suggest that miR-146a acts in T cells and B cells to prevent Tfh and GC B-cell accumulation.

### MiR-146a deficiency in T cells initiates Tfh-cell expansion

We next investigated whether accumulation of Tfh cells could occur independently of neighbouring *Mir146a*^−*/*−^ B cells and myeloid cells in the same mice. To this end, we transferred purified naïve CD4^+^ T cells from *Mir146a*^*+/+*^ or *Mir146a*^−*/*−^ OT-II TCR-transgenic mice into *Cd28*^−*/*−^ recipients, which were then immunized with OVA precipitated in alum and killed 7 days later. There was a statistically significant increase in the percentage of OVA-specific Vβ5^+^ Tfh cells and in the percentage of total GC B cells in mice that received *Mir146a*^−*/*−^ OT-II cells, although the magnitude of the effect was smaller than that observed in intact *Mir146a*^−*/*−^ mice (Fig. 3e), suggesting that non-T cells, including B cells and myeloid cells, enhance this effect.

In a separate experiment, four different combinations of *Mir146a*^*+/+*^ or *Mir146a*^−*/*−^ CD4^+^ T cells and B cells were co-transferred into *Rag1*^−*/*−^ mice, which were immunized 14 days later with sheep red blood cells (SRBCs; Fig. 3f). Similar to the OT-II transfer experiment, we found that in the presence of wild-type B cells and myeloid cells, miR-146a deficiency in T cells was sufficient to cause Tfh-cell accumulation, although the magnitude of the effect was again more modest than that observed in intact *Mir146a*^−*/*−^ mice (Fig. 3g). The presence of *Mir146a*^−*/*−^ B cells appeared to exacerbate the effect, but this did not reach statistical significance. Similar trends were observed for the increase in GC B cells (Fig. 3g). In summary, our data show that miR-146a deficiency in T cells is sufficient to initiate Tfh and GC B-cell accumulation, but T-cell-extrinsic factors contribute to exacerbate this phenotype.

### Contribution of IL-6 to Tfh and GC B-cell accumulation

In view of the reported increased proliferation of myeloid cells in 6- to 8-month-old *Mir146a*^−*/*−^ mice^20^,^34^, we asked whether expanded myeloid compartment could be one of the T-cell-extrinsic factors contributing to the Tfh and GC B-cell expansion. Assessments of 12- to 16-week-old mice, when the Tfh-cell accumulation is obvious (Supplementary Fig. 1), revealed no significant accumulation of myeloid cell subsets in *Mir146a*^−*/*−^ mice (Supplementary Fig. 2a).

**Figure 4:**
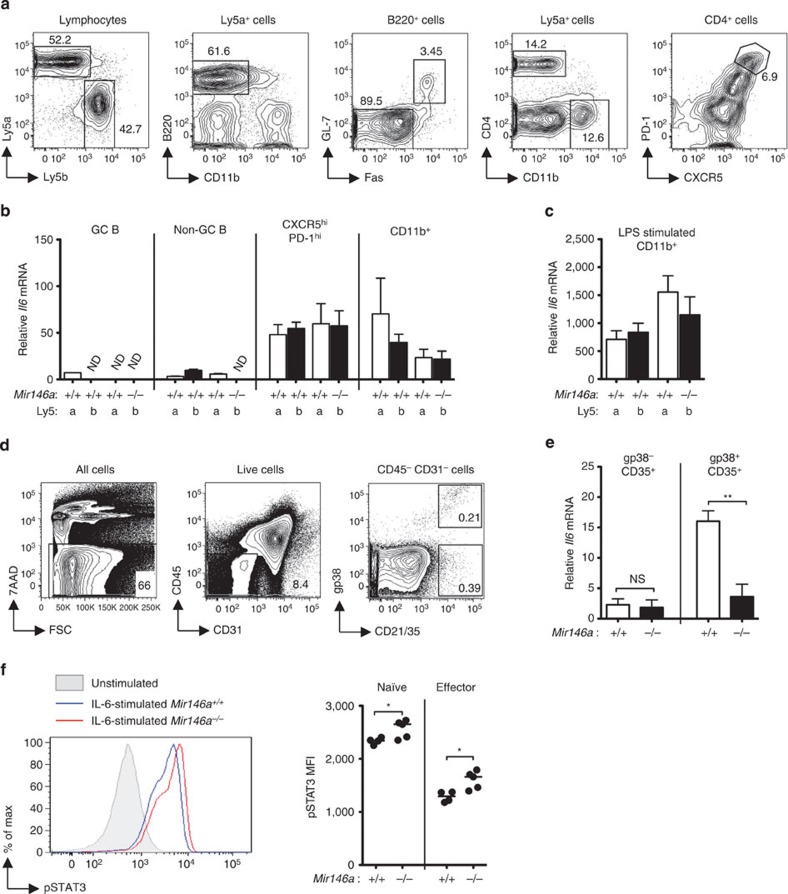
Contribution of IL-6 signalling to the Tfh and GC B-cell accumulation in miR-146a-deficient mice. (**a**–**c**) Similar expression of *Il6* mRNA between miR-146a-deficient (−/−) and -sufficient (+/+) cells in SRBC-immunized Ly5a^+^.*Mir146a*^*+/+*^: Ly5b^+^.*Mir146a*^−*/*−^ mixed bone marrow chimeras analyzed 14 weeks post reconstitution. (**a**) Flow cytometric contour plots showing the gating strategy used to isolate the different cell subsets. (**b**) Relative amounts of *Il6* mRNA in GC B (B220^+^ GL-7^+^ Fas^+^), non-GC B (B220^+^ GL-7^−^ Fas^−^), Tfh (CD4^+^ CXCR5^high^ PD-1^high^), and CD11b^+^ cells from Ly5a^+^ and Ly5b^+^ cells analyzed directly after isolation. ND=not detectable. The expression of *Il6* was normalized to β-actin. (**c**) Relative amounts of *Il6* mRNA in LPS-stimulated CD11b^+^ cells from the same set of chimeric mice as in **b**. (**d**,**e**) No increased expression of IL-6 in miR-146a-deficient follicular dendritic cells. (**d**) Flow cytometric contour plots showing the gating strategy used to isolate follicular dendritic cells. (**e**) Relative amounts of *Il6* mRNA in gp38^+^ and gp38^−^ FDCs (CD45^−^ CD31^−^ CD35^+^) from *Mir146a*^*+/+*^ or *Mir146a*^−*/*−^ mice. (**f**) Histograms and dot plots showing the amounts of STAT3 phosphorylation in naïve and effector CD4^+^ T cells following acute stimulation with recombinant mouse IL-6. Data are representative of at least two independent experiments. Heights of bar graph in **b**,**c**,**e** represent mean and error bar represents s.d. Each symbol in **f** represents one mouse and the horizontal bars represent the median values. Statistical significance was determined using unpaired Student’s *t*-test (**e**) or Mann–Whitney *U*-test (**f**). NS=not significant, *P<0.05, **P<0.01.

Myeloid cells and T cells have been reported to produce more IL-6 cytokine in 6- to 10-month-old miR-146a-null mice^20^,^23^. Since IL-6 is required for Tfh-cell formation^35^,^36^, we tested whether IL-6 production was elevated in 12- to 16-week-old *Mir146a*^−*/*−^ mice. No significant increase of IL-6 was observed in the serum of these mice (Supplementary Fig. 2b), and we also did not find increased amount of *Il6* transcripts in CD11c^high^ splenic dendritic cells (Supplementary Fig. 2b). Next, we used Ly5a^+^.*Mir146a*^*+/+*^: Ly5b^+^.*Mir146a*^−*/*−^ mixed bone marrow chimeras to investigate the possibility that during the course of an immune response, IL-6 might be expressed at higher levels in *Mir146a*^−*/*−^ GC B cells, Tfh cells or myeloid cells. Despite the expected cell-autonomous accumulation of *Mir146a*^−*/*−^ Tfh cells in the mixed chimeric mice (Supplementary Fig. 3), no differences in *Il6* mRNA expression were found between miR-146a-sufficient and miR-146a-deficient cells in any of the subsets examined (Fig. 4a–c). Finally, we tested the possibility that follicular dendritic cells (FDCs), which are of non-hematopoietic origin, expressed more IL-6 in the absence of miR-146a; it has been suggested that FDC-derived IL-6 is important for the late stage maintenance of Tfh cells during viral infection^35^. We isolated FDCs according to published protocols by gating on CD45^−^ CD31^−^ CD21/35^+^ cells from *Mir146a*^*+/+*^ or *Mir146a*^−*/*−^ mice. As both gp38-expressing^37^ and non-gp38-expressing^38^ FDCs have been identified, we separated these two cell subsets and measured the expression of IL-6 (Fig. 4d). Again, we did not find evidence of increased *Il6* transcripts in either gp38^+^ or gp38^−^ FDCs from miR-146-deficient mice (Fig. 4e). Nevertheless, a complete blockade of IL-6R using a previously reported dose of monoclonal antibody^35^ greatly reduced Tfh-cell accumulation in *Mir146a*^−*/*−^ mice (Supplementary Fig. 4), indicating that miR-146a-driven Tfh-cell accumulation is still dependent on IL-6-mediated signalling.

Intriguingly, we observed enhanced IL-6-induced STAT3 phosphorylation in naïve and effector CD4^+^ T cells from *Mir146a*^−*/*−^ mice (Fig. 4f). Lack of increased STAT1 phosphorylation in parallel assays (Supplementary Fig. 5) argued against STAT1 overexpression being the cause of this enhanced sensitivity to IL-6. Collectively, these results suggest that increased IL-6 expression is probably not the main cause of the Tfh-cell accumulation in miR-146a-deficient mice; rather, the increased sensitivity to IL-6 signalling is a more likely contributor to the phenotype.

### MiR-146a represses multiple Tfh-cell mRNAs

To uncover mRNA targets of miR-146a in T cells, we performed a microarray analysis on sorted naïve CD4^+^ T cells from *Mir146a*^*+/+*^ and *Mir146a*^−*/*−^ mice. A number of differentially expressed genes were identified and for further validation, we selected putative targets based on meeting two of the following three criteria: (1) upregulated mRNA expression in *Mir146a*^−*/*−^ T cells assessed by global gene expression profiling, (2) known roles in Tfh and/or GC biology and (3) the presence of target sites for miR-146a seed region within their 3′ untranslated region (UTR). The selected mRNA targets were *Stat1*, *Cxcr4*, *Notch1*, *Slamf1*, *Slamf6*, *Cd84*, *Icos*, *Il6st* and *Il21r* (putative miR-146a binding sites are shown in Supplementary Fig. 6; previously validated target sites in *Cxcr4, Notch1* and *Stat1* are not shown).

**Figure 5:**
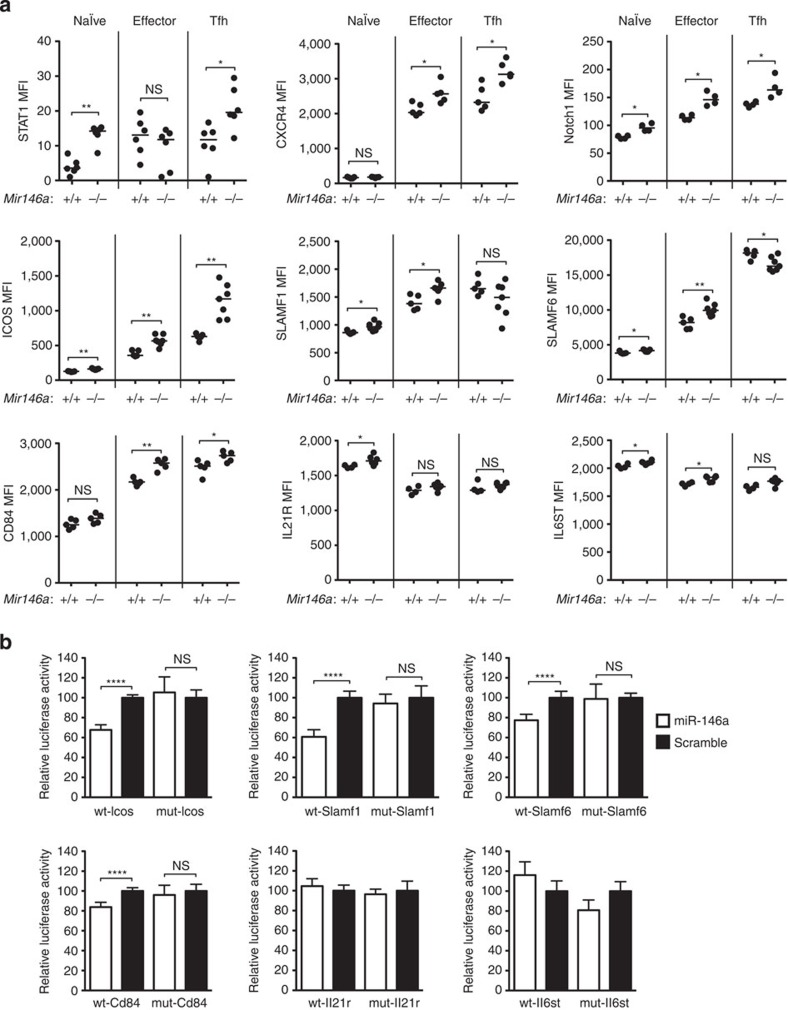
MiR-146a directly represses multiple Tfh-cell mRNAs. (**a**) Geometric mean fluorescence intensity (MFI) of STAT1, CXCR4, Notch1, ICOS, SLAMF1, SLAMF6, CD84, IL-21R and IL6ST on naïve (CD4^+^ CD44^low^), effector (CD4^+^ CD44^high^) and Tfh (CD4^+^ CXCR5^high^ PD-1^high^ Foxp3^−^) cells from mice lacking miR-146a (−/−) or wild-type littermates (+/+). Each symbol represents one mouse and the horizontal bars represent the median values. Data are representative of at least two independent experiments. Statistical significance was determined using a Mann–Whitney *U*-test. (**b**) Effects of miR-146a (open bars) and scramble (negative control) RNA (filled bars) expression on luciferase reporter constructs containing putative miR-146a target sites as relative luciferase activity (normalized to the *Renilla* control). Sequence specificity of miRNA–mRNA binding was confirmed by introducing mutations to the predicted target sites at regions complementary to the miR-146a seed region. Relative luciferase activity of cells transfected with miR-146a is expressed as a percentage of that transfected with the scramble RNA. Data are representative of at least two independent experiments. The heights of the bars represent the mean, and the error bars represent the s.d. of six technical replicates. Statistical significance was determined using unpaired Student’s *t*-test. NS, not significant, *P<0.05, **P<0.01, ****P<0.0001.

Next we assessed by flow cytometry whether the proteins encoded by these putative RNA targets were upregulated in miR-146a-deficient T cells. STAT1, a validated target of miR-146a in Tregs^24^ and an important signalling intermediate and transcription factor downstream of IL-6, IL-12, IL-21 and IL-27, was upregulated in *Mir146a*^−*/*−^ naïve CD4^+^, Tfh cells (Fig. 5a) and, as shown previously^24^, in Tregs (Supplementary Fig. 7). The validated miR-146a targets CXCR4 (ref. 39), known to regulate dark zone versus light zone partitioning in GCs^5^,^40^,^41^, and Notch1 (ref. 42), shown to promote Tfh-cell differentiation^43^, were both upregulated in *Mir146a*^−*/*−^ effector CD4^+^, Tfh cells (Fig. 5a) and in Tregs (Supplementary Fig. 7). ICOS is required for providing costimulatory signals for optimal Tfh and GC B-cell differentiation^44^,^45^,^46^,^47^ and for persistent directional motility and follicular recruitment of activated CD4^+^ T cells^48^ and failed *Icos* mRNA repression is associated with accumulation of Tfh cells in lupus-prone Roquin-1/2 mutant mice^6^,^19^,^49^. We observed ICOS overexpression in *Mir146a*^−*/*−^ naïve, effector CD4^+^, Tfh cells (Fig. 5a) and Tregs (Supplementary Fig. 7). Members of the SLAM family of receptors (SLAMF1, SLAMF6 and CD84) mediate prolonged interactions between activated antigen-specific T and B cells and signal via the recruitment of SAP to promote terminal Tfh-cell differentiation^50^. SLAMF1, SLAMF6 and CD84 were upregulated in *Mir146a*^−*/*−^ naïve and effector CD4^+^ T cells compared with their wild-type counterparts (Fig. 5a). CD84 was upregulated in *Mir146a*^−*/*−^ Tfh cells (Fig. 5a), and SLAMF1 and CD84 were also upregulated in *Mir146a*^−*/*−^ Tregs (Supplementary Fig. 7). IL-21R and IL6ST are subunits of receptors for the Tfh-cell-inducing cytokines IL-21 and IL-6, respectively. IL-21R was upregulated in *Mir146a*^−*/*−^ naïve (Fig. 5a) and Tregs (Supplementary Fig. 7), while IL6ST was upregulated in *Mir146a*^−*/*−^ naïve and effector CD4^+^ T cells (Fig. 5a), but not in Tfh cells (Fig. 5a) or Tregs (Supplementary Fig. 7).

The putative targets that had not been previously validated (*Slamf1*, *Slamf6*, *Cd84*, *Icos*, *Il6st* and *Il21r*) were further tested for direct repression by miR-146a using luciferase reporter assays. The predicted target sequence from *Stat1* revealed a 25% repression (Supplementary Fig. 8), comparable to that reported by Rudensky and co-workers^24^. Similar repression was observed using luciferase reporters containing the minimal target sequences from *Icos*, *Slamf1*, *Slamf6* or *Cd84.* All of these targets were specifically repressed by miR-146a, but not by a ‘scramble’ miRNA control (a commercially available, random sequence miRNA mimic; Fig. 5b). Luciferase reporter constructs containing mutations in the sequences recognized by the miR-146a seed region were no longer repressed by miR-146a. This not only validated the sequence specificity of the miRNA–mRNA binding but also mapped the miRNA recognition sequence in the 3′UTR (Fig. 5b). By contrast, luciferase reporter constructs containing the predicted target sequences from *Il21r* and *Il6st* were not repressed by miR-146a (Fig. 5b). Hence, *Icos*, *Slamf1*, *Slamf6* and *Cd84*, but not *Il21r* and *Il6st*, are likely to be novel direct targets of miR-146a.

**Figure 6:**
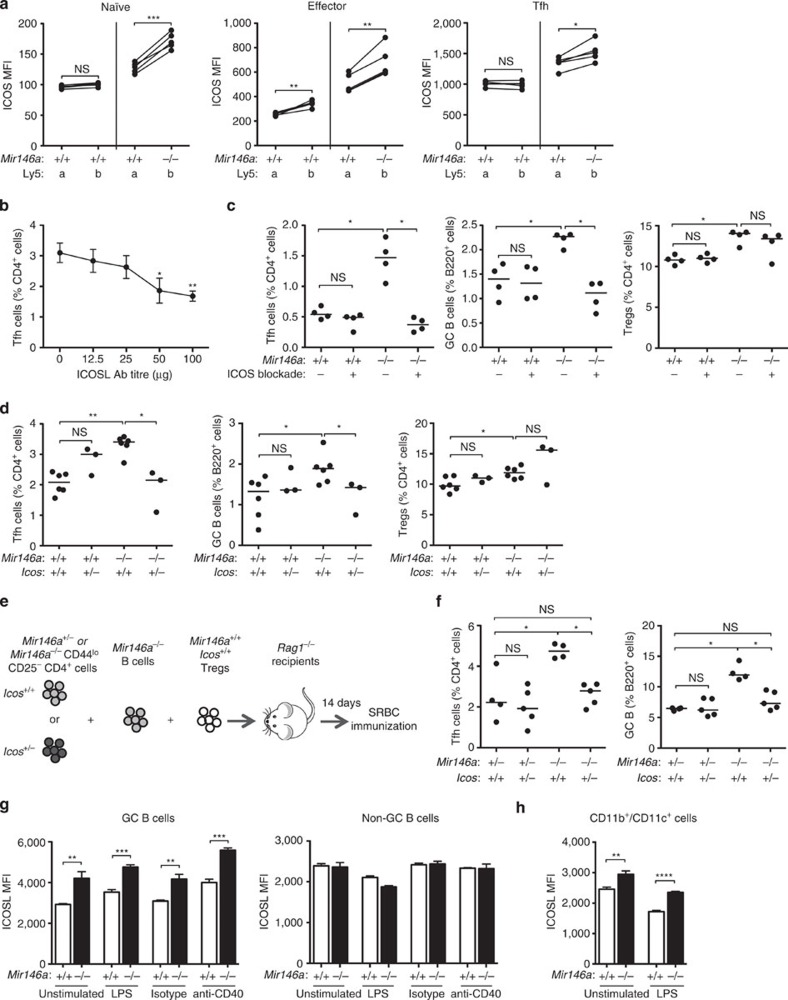
Increased ICOS and ICOSL expression is likely to be the major driver of Tfh-cell accumulation in the miR-146a-deficient mice. (**a**) Geometric mean fluorescence intensity (MFI) of ICOS on naïve, effector CD4^+^ and Tfh cells from *Mir146a*^*+/+*^: *Mir146a*^−*/*−^ and *Mir146a*^*+/+*^: *Mir146a*^*+/+*^ mixed bone marrow chimeras 16 weeks post reconstitution. (**b**) Percentages of Tfh cells in wild-type mice immunized with sheep red blood cells (SRBC) and treated with different amounts of anti-ICOSL blocking antibody on days 0, 3 and 6 post immunization. Each symbol represents the mean and error bars represent the s.d. of three biological replicates. (**c**) Percentages of Tfh, GC B and Treg cells in 9-week-old wild-type (+/+) and miR-146a-deficient mice (−/−) treated with 25 μg anti-ICOSL blocking antibody or PBS control on day 0, 3 and 6 following SRBC immunization. (**d**) Percentages of Tfh, GC B and Treg cells in 9-week-old *Mir146a*^*+/+*^ *Icos*^*+/+*^, *Mir146a*^*+/+*^ *Icos*^*+/*−^, *Mir146a*^−*/*−^ *Icos*^*+/+*^, *Mir146a*^−*/*−^ *Icos*^*+/*−^ mice following SRBC immunization. (**e**,**f**) Adoptive transfer of purified naïve (CD44^low^ CD25^−^) CD4^+^ T cells from either *Mir146a*^*+/*−^ *Icos*^*+/+*^, *Mir146a*^*+/*−^ *Icos*^*+/*−^, *Mir146a*^−*/*−^ *Icos*^*+/+*^ or *Mir146a*^−*/*−^ *Icos*^*+/*−^, together with *Mir146a*^−*/*−^ *Icos*^*+/+*^ B cells and *Mir146a*^*+/+*^ *Icos*^*+/+*^ Tregs into *Rag1*^−*/*−^ recipients according to the strategy shown in **e**. The recipient mice were immunized with SRBC 14 days after transfer and killed 7 days post immunization. (**f**) Percentages of Tfh and GC B cells in *Rag1*^−*/*−^ recipient mice following adoptive transfer and SRBC immunization. (**g**,**h**) ICOSL MFI on GC B (**g**, left), non-GC B (**g**, right) and a mixture of CD11b^+^ and CD11c^+^ cells (**h**) from SRBC-immunized wild-type (+/+) or miR-146a-deficient (−/−) mice following stimulation with LPS, anti-CD40 antibody or isotype control. Heights of bars represent the mean and error bars represent s.d. of three technical replicates. Each symbol represents one mouse and the horizontal bars represent the median values. Data are representative of at least two independent experiments. Statistical significance was determined using paired Student’s *t*-test (**a**), unpaired Student’s *t*-test (**b**,**g**,**h**) or Mann–Whitney *U*-test (**c**,**d**,**f**). NS, not significant, *P<0.05, **P<0.01, ***P<0.001, ****P<0.0001.

Luciferase assays reveal targets of miRNAs when the miRNA is overexpressed in HEK293T cells. To assess which of the above targets are physiologically relevant and a direct consequence of miR-146a regulation in CD4^+^ T cells (naïve, effectors, Treg and Tfh cells), we assessed their protein expression in Ly5a^+^*.Mir146a*^*+/+*^: Ly5b^+^.*Mir146a*^−*/*−^ mixed bone marrow chimeras. For this, the geometric mean fluorescence intensity was compared between Ly5a^+^ and Ly5b^+^ cells for each target. Of all targets, the only one that appeared to be controlled cell autonomously by miR-146a in all CD4^+^ subsets examined (including Tfh cells) was ICOS (Fig. 6a). None of the other targets was cell autonomously upregulated in Tfh cells, but some cell-autonomous upregulation was observed in naïve CD4^+^ T cells (Supplementary Fig. 9: CXCR4, SLAMF1, SLAMF6 and CD84), effector CD4^+^ T cells (Supplementary Fig. 9: CXCR4) and Tregs (Supplementary Fig. 10: CXCR4, SLAMF1, CD84 and STAT1). As expected from the luciferase assay results, neither IL6ST nor IL-21R were upregulated in a cell-intrinsic manner in *Mir146a*^−*/*−^ T cells (Supplementary Figs 9 and 10). Collectively, our data suggest that miR-146a acts in T cells to directly repress the expression of *Icos*, *Slamf1*, *Slamf6*, *Cd84* and *Cxcr4*, and of these, the only cell-autonomous effect in Tfh cells is exerted on *Icos*.

### Increased ICOS signalling in T cells causes Tfh-cell accumulation

In light of the profound cell-autonomous control of ICOS by miR-146a and the known roles of ICOS in promoting Tfh-cell differentiation^19^,^48^,^51^, we set out to formally test the contribution of excessive ICOS signalling to the expansion of Tfh cells in the absence of miR-146a. To this end, we first titrated anti-ICOSL antibody treatment in mice in search of a dose sufficiently low so as to partially suppress ICOS signalling without abolishing Tfh-cell formation in wild-type mice. Injections of 25 μg of anti-ICOSL antibody given every 3 days to wild-type mice after immunization with SRBCs had no significant effect on wild-type Tfh cells (Fig. 6b). We then used this anti-ICOSL treatment regime on 9-week-old *Mir146a*^*+/+*^ and *Mir146a*^−*/*−^ mice immunized with SRBC. This treatment completely and consistently corrected the accumulation of Tfh and GC B cells in *Mir146a*^−*/*−^ mice and still had no effect on wild-type mice (Fig. 6c). Of note, the increase in Tregs seen in *Mir146a*^−*/*−^ mice was not consistently corrected by the anti-ICOSL treatment (Fig. 6c and Supplementary Fig. 11).

We also used a genetic approach to reduce but not eliminate ICOS expression in *Mir146a*^−*/*−^ mice. *Icos* hemizygosity was achieved by crossing *Mir146a*^−*/*−^ to *Icos*^−*/*−^ mice to generate *Mir146a*^−*/*−^ *Icos*^*+/*−^ mice. As seen with the antibody blockade, lowering *Icos* gene dose reduced the percentage of Tfh and GC B cells in *Mir146a*^−*/*−^ *Icos*^*+/*−^ mice to the amount seen in wild-type *Mir146a*^*+/+*^ *Icos*^*+/+*^ mice (Fig. 6d). Importantly, *Icos* hemizygosity did not rescue Treg-cell accumulation in mice lacking miR-146a (Fig. 6d), suggesting that dysregulation of Treg number is not the cause of Tfh-cell accumulation. Thus, our data indicate that overexpression of ICOS is likely to be a prominent contributor to the Tfh-cell accumulation in *Mir146a*^−*/*−^ mice.

To exclude the possibility that the observed effect of *Icos* gene dose reduction was a consequence of modulating ICOS expression on Tregs, we co-transferred sorted naïve (CD44^low^ CD25^−^) CD4^+^ T cells from *Mir146a*^*+/*−^ or *Mir146a*^−*/*−^ mice, which were either *Icos*^*+/+*^ or *Icos*^*+/*−^, with *Mir146a*^−*/*−^ *Icos*^*+/+*^ B cells and *Mir146a*^*+/+*^ *Icos*^*+/+*^ Tregs into *Rag1*^−*/*−^ recipient mice (Fig. 6e). *Mir146a*^*+/*−^ mice behaved identically to *Mir146a*^*+/+*^ with no evidence of spontaneous Tfh or GC B-cell accumulation (Supplementary Fig. 12a) or ICOS overexpression (Supplementary Fig. 12b). Fourteen days after adoptive transfer, T cells in all experimental groups showed comparable activation status as measured by the proportion of cells upregulating CD44 or CD25 (Supplementary Fig. 13). Following SRBC immunization, recipients of *Mir146a*^−*/*−^ *Icos*^*+/*−^ T cells had a ~50% reduction in the percentage of Tfh and GC B cells compared with those that received *Mir146a*^−*/*−^ *Icos*^*+/+*^ T cells (Fig. 6f). Furthermore, halving the gene dose of *Icos* on *Mir146a*^−*/*−^ T cells virtually corrected the Tfh-cell accumulation caused by the lack of miR-146a in T cells: there was no statistically significant difference between the *Mir146a*^*+/*−^ *Icos*^*+/+*^ and *Mir146a*^−*/*−^ *Icos*^*+/*−^ groups. These results suggest that *Icos* overexpression in Tfh-cell precursors is indeed responsible, at least in part, for the Tfh-cell accumulation in miR-146a-deficient mice.

### MiR-146a limits ICOSL in GC B cells and dendritic cells

The mixed bone marrow chimera results showed that despite the increase in total follicular T cells in the Ly5a^+^*.Mir146a*^*+/+*^: Ly5b^+^.*Mir146a*^−*/*−^ chimeras, only the Ly5b^+^.*Mir146a*^−*/*−^ GC B cells, and not the Ly5a^+^.*Mir146a*^*+/+*^ B cells, expanded (Fig. 3c,d). One of the plausible explanations is that miR-146a co-ordinately regulates a receptor–ligand pair in Tfh and GC B cells, so overexpression of both T-cell-expressed receptor and GC B-cell ligand (or *vice versa*) are required for *Mir146a*^−*/*−^ GC B cells to accumulate. Since our data revealed ICOS as the most prominent target of miR-146a in Tfh cells, and T-cell-expressed ICOS interacts with B-cell-expressed ICOS ligand (ICOSL) at different stages of Tfh-cell and GC formation^48^,^51^, we assessed whether miR-146a also regulates ICOSL in B cells. Although we did not find any predicted miR-146a binding site in *Icosl* 3′UTR, previous studies have shown that *Icosl* transcription is regulated by NF-κB signalling^52^, which is partly controlled by miR-146a (refs 20, 21, 23).

To test whether miR-146a controls ICOSL expression in B cells, we sorted total B cells from *Mir146a*^*+/+*^ and *Mir146a*^−*/*−^ mice, stimulated them in culture for 8 h using lipopolysaccharides (LPS) or anti-CD40 antibody, and compared ICOSL expression on either GC or non-GC B cells. No differences in ICOSL expression were observed in non-GC B cells under any conditions (Fig. 6g). On the other hand, the expression of ICOSL was consistently higher in *Mir146a*^−*/*−^ GC B cells—known to be dependent on NF-κB signalling^53^,^54^,^55^—across all conditions (Fig. 6g). An increase in ICOSL expression was also observed in miR-146a-deficient CD11b^+^ and CD11c^+^ cells following *in vitro* culture in the absence or presence of LPS (Fig. 6h). These results suggest that overexpression of ICOSL on GC B cells and myeloid/dendritic cells are likely to enhance the effects of increased ICOS expression on Tfh-cell accumulation. They also suggest that ICOSL overexpression may be required for *Mir146a*^−*/*−^ GC B-cell expansion when ICOS is concomitantly overexpressed in T cells.

Finally, we explored the role of other miR-146a targets that may contribute to the Tfh-cell accumulation. To this end, we investigated the consequences of genetic manipulation of STAT1 that has known Tfh-cell-promoting effects^51^ and SLAMF1. *Mir146a*^−*/*−^ mice were crossed with mice carrying a loss-of-function mutation in the *Stat1* gene (*Stat1*^*fae*^; MGI:3611892) to generate *Mir146a*^−*/*−^ *Stat1*^*fae/+*^ mice (Supplementary Fig. 14). The percentages of Tfh and GC B cells in *Mir146a*^−*/*−^ *Stat1*^*fae/+*^ mice were significantly lower than those in the *Mir146a*^−*/*−^ *Stat1*^*+/+*^ mice, but remained about eightfold higher than those seen in wild-type mice (Supplementary Fig. 14). This result suggests that STAT1 contributes to the Tfh-cell accumulation in *Mir146a*^−*/*−^ mice, perhaps by crippling Treg control of Tfh responses or by actions within Tfh cells and/or other cells. We also studied the effect of increased *Slamf1* copy number in Tfh-cell formation using NOD.*Nkrp1b*.Tg(*Slamf1*)1 (SLAM A—6 copies of *Slamf1*) and NOD.*Nkrp1b*.Tg (*Slamf1*)2 (SLAM B—44 copies^56^) mice (Supplementary Fig. 15). No differences were identified in either Tfh-cell or GC formation. Thus, in addition to the regulation of ICOS, miR-146a also represses ICOSL and STAT1 expression, which collectively may contribute to the accumulation of Tfh cells in mice lacking miR-146a.

## Discussion

We have shown that miR-146a, a microRNA highly expressed in Tfh cells, limits Tfh-cell number. Indeed, in the absence of miR-146a, there was spontaneous expansion of Tfh and GC B cells. MiR-146a emerges as the first miRNA to negatively regulate Tfh-cell accumulation. It therefore opposes the action of the miR-17~92 cluster, recently shown to promote Tfh-cell differentiation by repressing *Pten, Phlpp2* and *Rora* mRNAs^57^,^58^.

Our luciferase assays, together with published reports, have shown that ectopic expression of miR-146a can lead to repression of multiple Tfh-cell-expressed mRNAs, including *Icos, Slamf1, Slamf6, Cd84*, *Stat1, Cxcr4* and *Notch1*. Although all of the above targets were upregulated in T cells from miR-146a-deficient mice, ICOS was the only target upregulated cell autonomously in Tfh cells and the most robustly upregulated across all T-cell subsets examined. Strikingly, dampening ICOS-mediated signalling in *Mir146a*^−*/*−^ mice, either by generating *Mir146a*^−*/*−^ *Icos*^*+/*−^ mice or by injecting a suboptimal dose of anti-ICOSL antibody, completely prevented miR-146a-driven Tfh-cell accumulation. Furthermore, adoptive transfer experiments in which only non-Tregs lacked one allele of *Icos* demonstrated that repressing ICOS signalling in Tfh-cell precursors themselves, rather than in Tregs, is required to control Tfh-cell number. This is consistent with previous reports showing that cell-autonomous *Icos* mRNA overexpression due to defective Roquin function contributes to excessive Tfh-cell number^19^.

We have also found that miR-146a acts cell autonomously in GC B cells to limit their number. Increased expression of ICOSL on GC B cells is a plausible contributor to their accumulation in the absence of miR-146a, and may explain why despite the striking increase of *Mir146a*^−*/*−^ Tfh cells in *Mir146a*^*+/+*^: *Mir146a*^−*/*−^ chimeras only *Mir146a*^−*/*−^ GC B cells expanded. Limiting ICOSL signalling is likely to be important to prevent excessive B-cell activation. Indeed, mice overexpressing soluble ICOSL-Fc developed hypergammaglobulinemia and plasmacytosis^59^. Our results suggest that concomitant control of ICOS on Tfh cells and ICOSL on GC B may be necessary to control the number of GC B cells.

In addition to ICOS–ICOSL regulation, miR-146a-mediated repression of other T-cell-expressed targets might also contribute to limit Tfh cells. Our data suggest STAT1 is one such target: lowering the dose of STAT1 had a significant corrective effect on Tfh-cell accumulation. This effect is likely to, at least in part, be a consequence of STAT1 overexpression in Treg cells, including Tfr cells^15^, leading to impaired repressive ability^24^. In addition, STAT1 is known to function downstream of several Tfh-inducing cytokines, including IL-6, IL-12, IL-21 and IL-27 (refs 3, 51, 60), thus STAT1 overexpression may enhance the sensitivity of miR-146a-deficient T cells to stimulation by these cytokines and increase their propensity to differentiate along the Tfh lineage. Nevertheless, we did not observe an increase in IL-6-induced STAT1 phosphorylation in *Mir146a*^−*/*−^ T cells. Another candidate that may contribute to the Tfh-cell accumulation in miR-146a-deficient mice is IL6ST. We found a subtle increase in IL6ST expression on *Mir146a*^−*/*−^ T cells, although our luciferase assay data indicate that it is unlikely to be a direct target of miR-146a. We speculate that increased IL6ST expression in *Mir146a*^−*/*−^ T cells might cause the observed heightened sensitivity of these T cells to IL-6 signalling as measured by STAT3 phosphorylation.

Although this work has focused on identifying Tfh cell-autonomous targets of miR-146a, the milder phenotype observed when only T cells lack miR-146a suggests that T-cell extrinsic factors also contribute to the accumulation of Tfh cells in animals lacking miR-146a. IL-6, which was increased in aged miR-146a-deficient mice^20^,^23^, did not appear to be upregulated in any of the cell types examined in 12- to 16-week-old mice, although a role of IL-6 derived from other haematopoietic cells and/or non-haematopoietic cells cannot be completely excluded. Furthermore, given the significant effect of IL-6R blockade treatment, it is possible that *Mir146a*^−*/*−^ T cells or myeloid cells do produce increased IL-6 protein *in vivo* that would not have been detected by our transcriptional studies. On the other hand, antigen-presenting cells that are typically CD11c^+^ and/or CD11b^+^ did express increased amounts of ICOSL, which may enhance T-cell priming and exacerbate Tfh-cell accumulation in *Mir146a*^−*/*−^ mice^61^. Tfr cells are required to control Tfh cells and GC B cells^15^,^16^,^17^ and have been shown to derive from Foxp3^+^ thymic Tregs, which are less functional in *Mir146a*^−*/*−^ mice due to STAT1 overexpression^24^. Thus, despite their accumulation, it is likely that Tfr cells are also defective in *Mir146a*^−*/*−^ mice and this may also exacerbate the spontaneous Tfh-cell accumulation. In addition, increased expression of the costimulatory molecules CD80 (ref. 62) and CD86 (ref. 63) and the reported enhanced sensitivity of T cells to antigen stimulation^21^ due to dysregulated NF-κB activation may also contribute to the Tfh-cell expansion. It has been reported previously that strong TCR signalling can skew the differentiation pathway of naïve CD4^+^ T cells towards the Tfh lineage^64^,^65^. Collectively, all of these miR-146a-mediated effects in various cell types are likely to act cooperatively to cause the accumulation of Tfh cells.

In conclusion, this report identifies miR-146a as an important negative regulator of Tfh and GC B-cell accumulation. It is likely that this contributes to maintaining immunological tolerance and preventing the autoimmune phenotypes observed in mice lacking this microRNA. These findings on miR-146a-mediated control of Tfh and GC B-cell number may therefore improve our understanding of human autoimmunity and inflammatory syndromes.

*Note added in proof*: A role for miR-146a in repressing miR-155-induced Tfh-cell accumulation was reported while this work was under consideration^66^.

## Methods

### Mice and immunizations

The mice used were C57BL/6 (B6), B6.Ly5a, B6.*Rag1*^−*/*−^, B6.*Cd28*^−*/*−^, B6.OT-II, B6.*Mir146a*^−*/*−^ (ref. 20), B6.*Stat1*^*fae/+*^, B6.*Icos*^*+/*−^ (Australian Phenomics Facility, ANU), NOD.*Nkrp1b*.Tg(*Slamf1*)1 (ref. 56) and NOD.*Nkrp1b*.Tg(*Slamf1*)2 (ref. 56; Immunogenetics Research Facility, James Cook University). Male and female mice were used in experiments at approximately equal ratio between the wild-type/untreated and mutant/treated groups. *Mir146a*^−*/*−^ mice were provided by David Baltimore (Caltech, USA). *Stat1-face* (*Stat1*^*fae*^) mice were derived from an N-ethyl N-nitrosourea mutagenesis screen^67^ and kindly donated by Christopher C. Goodnow (ANU, Australia). *Mir146a*^−*/*−^, *Mir146a*^−*/*−^ *Stat1*^*fae/+*^, NOD.*Nkrp1b*.Tg(*Slamf1*)1 (ref. 56) and NOD.*Nkrp1b*.Tg (*Slamf1*)2 (ref. 56) mice were taken down at 12 to 16 weeks old unless otherwise stated. All mice were housed under a specific pathogen-free environment. All experiments were approved by the Australian National University Animal Experimentation Ethics Committee and the James Cook University Animal Ethics Committee. To generate thymus-dependent GC responses, where mentioned, mice were immunized i.v. with 2 × 10^8^ SRBCs (Applied Biological Products Management, Australia) and were taken down at day 7 post immunization.

### Human tonsils

Human tonsils were obtained from consenting patients undergoing routine tonsillectomy at the Canberra Hospital (Canberra, Australia) with the approval from the institutional human research ethics committee. Tonsillar cells were isolated by mechanical disruption and Ficoll-Paque density gradient centrifugation.

### Adoptive transfers

To study the kinetics of miR-146a expression in a thymus-dependent GC response, 5 × 10^5^ sorted naïve OT-II CD4^+^ T cells were adoptively transferred into Ly5a^+^ wild-type B6 recipient mice via i.v. injection. To limit miR-146a deficiency to T cells, 1 × 10^5^ sorted naïve *Mir146a*^−*/*−^ OT-II or *Mir146a*^*+/+*^ OT-II T cells were adoptively transferred into *Cd28*^−*/*−^ recipient mice. The recipients were immediately immunized i.p. with 100 μg OVA (Sigma) precipitated in alum. To study the contributions of miR-146a deficiency in T and B cells, 4 × 10^6^ CD4^+^ T cells and 6 × 10^6^ B cells from *Mir146a*^*+/+*^ or *Mir146a*^−*/*−^ mice were adoptively transferred into *Rag1*^−*/*−^ mice. The recipients were immunized with SRBC 14 days after transfer and they were killed 7 days post immunization. In addition, to examine the contribution of ICOS overexpression specifically in Tfh-cell precursors to the Tfh-cell accumulation, 7.5 × 10^5^ CD44^low^ CD25^−^ CD4^+^ T cells from either *Mir146a*^*+/*−^ *Icos*^*+/+*^, *Mir146a*^*+/*−^ *Icos*^*+/*−^, *Mir146a*^−*/*−^ *Icos*^*+/+*^ or *Mir146a*^−*/*−^ *Icos*^*+/*−^ donor mice were co-transferred with 3 × 10^6^ *Mir146a*^−*/*−^ *Icos*^*+/+*^ B cells and 7.5 × 10^4^ *Mir146a*^*+/+*^ *Icos*^*+/+*^ Tregs into *Rag1*^−*/*−^ recipients. The recipients were bled (to assess the activation status of T cells) at day 14 following adoptive transfer and immediately immunized with SRBC. The mice were then killed 7 days post immunization.

### Antibodies

Antibodies and streptavidin conjugates used for flow cytometry were (respective vendors, catalogue numbers and dilutions at which they were used are shown in parentheses): anti-mouse Ly5a-Pacific Blue (Biolegend, 110722, 1:200), Ly5a-Alexa Fluor 700 (BD, 561235, 1:200), Ly5b-Alexa Fluor 700 (BD, 560693, 1:200), Ly5b-PerCP Cy5.5 (BD, 552950, 1:200), CD4-Alexa Fluor 700 (BD, 557956, 1:800), CD4-APC Cy7 (BD, 552051, 1:800), B220-PerCP (BD, 553093, 1:800), CXCR5-biotin (BD, 551960, 1:50), PD-1-PE (BD, 551892, 1:100), PD-1-Brilliant Violet 421 (BD, 562584, 1:400), CD25-Alexa Fluor 647 (BD, 563598, 1:100), CD44-APC (BD, 559250, 1:400), CD44-Alexa Fluor 700 (BD, 560567, 1:400), CD44-PE CF594 (BD, 562464, 1:400), Foxp3-FITC (eBioscience, 11-5773-82, 1:400), Foxp3-eFluor 450 (eBioscience, 48-5773-82, 1:400), GL-7-FITC (BD, 553666, 1:400), Fas-PE Cy7 (BD, 557653, 1:400), Vβ5.1/5.2-FITC (BD, 562087, 1:400), ICOSL-PE (Biolegend, 107405, 1:400), CD11b-APC Cy7 (BD, 557657, 1:200), CD11c-PerCP Cy5.5 (BD, 560584, 1:200), IFN-γ-FITC (BD, 554411, 1:400), IL-4-PE Cy7 (BD, 560699, 1:100), IL-17A-Alexa Fluor 700 (BD, 560820, 1:100), STAT1 (pY701)-Pacific Blue (BD, 560310, 1:5), STAT3 (pY705)-PE (BD, 562072, 1:20), STAT1-Alexa Fluor 647 (BD, 558560, 1:40), CXCR4-biotin (BD, 551968, 1:200), Notch1-PE (BD, 552768, 1:200), ICOS-PE (BD, 552146, 1:200), SLAMF1-PE (BD, 562651, 1:200), SLAMF6-Alexa Fluor 647 (BD, 561547, 1:100), CD84-PE (Biolegend, 122806, 1:200), IL-21R-APC (Biolegend, 131910, 1:100), gp130/IL6ST-APC (R&D Systems, FAB4681A-100, 1:100), CD31-PE (BD, 561073, 1:200), gp38-Alexa Fluor 488 (eBioscience, 53-5381-82, 1:200), CD21/35-biotin (BD, 562796, 1:200), anti-human CD4-APC Cy7 (BD, 557871, 1:800), CXCR5-Alexa Fluor 647 (BD, 558113, 1:100), PD-1-PE (eBioscience, 12-9969-42, 1:200), CD25-PE Cy7 (Biolegend, 356108, 1:100), CD44-Alexa Fluor 700 (BD, 560567, 1:400), and streptavidin-PE Cy7 (BD, 557598, 1:800). Monoclonal blocking antibody against ICOSL (clone MIL-5733)^68^ was provided by A.H. (DRFZ, Germany) and blocking antibody against IL-6R (catalogue number BE0047, 150 μg per mouse) was purchased from BioXCell. Mouse anti-CD40 (catalogue number BE0016-2, 10 μg ml^−1^) antibody was also purchased from BioXCell.

### Bone marrow chimeras

Chimeras were generated by sub-lethally irradiating *Rag1*^−*/*−^ recipient mice (500 cGy, X-rad) and reconstituting their immune system by transferring 4 × 10^6^ bone marrow cells from donor mice. Chimeric mice were taken down 16 weeks post reconstitution unless otherwise stated.

### Cell isolation and stimulation

Single-cell suspensions were prepared from spleens of unimmunized and/or immunized mice. Suspensions were prepared in RPMI 1640 media (Gibco) supplemented with 10% heat-inactivated foetal calf serum (FCS), 100 U penicillin–streptomycin (Gibco), 2 mM L-Glutamine (Gibco), 10 mM HEPES (Sigma), 0.1 mM non-essential amino acids (Gibco), 1 mM sodium pyruvate (Sigma), 55 μM 2-mercaptoethanol (Gibco) by mechanical disruption and gentle pipetting through 70 μm nylon mesh filters (BD Biosciences). For cytokine staining (IFN-γ, IL-4 and IL-17), cells were cultured for 4 h in a 37 °C/5% CO_2_ incubator with 50 ng ml^−1^ PMA (Sigma) and 1 μM ionomycin (Sigma) in the presence of Golgi Stop (BD Biosciences). For ICOSL induction, B220^+^ cells and combined CD11b^+^/CD11c^+^ cells were isolated from SRBC-immunized *Mir146a*^*+/+*^ or *Mir146a*^−*/*−^ mice by negative and positive selection, respectively, using MACS columns (Miltenyi Biotec) according to the manufacturer’s instructions. About 5 × 10^5^ B cells or CD11b^+^/CD11c^+^ cells were cultured per well in a 96-well plate (Corning) with different conditions: complete RPMI only (unstimulated), 1 μg ml^−1^ LPS from *Escherichia coli* (Sigma), 10 μg ml^−1^ rat IgG2a isotype control antibody (BioXCell) or 10 μg ml^−1^ rat anti-mouse CD40 antibody (BioXCell) for 8 h in a 37 °C/5% CO_2_ incubator. To induce IL-6 expression, purified CD11b^+^ cells from mixed bone marrow chimeras were stimulated overnight with 100 ng ml^−1^ LPS (Sigma) in a 37 °C/5% CO_2_ incubator. To study the responsiveness of T cells to IL-6 signalling, total splenocytes were stimulated with recombinant mouse IL-6 (20 ng ml^−1^) for 15 min in a 37 °C/5% CO_2_ incubator and levels of STAT3 phosphorylation were analyzed according to published protocols^69^.

### Flow cytometry

For lymphocyte analysis, single-cell suspensions were prepared as described in the previous section. For myeloid cell-population analysis, spleens were treated with Collagenase P (Roche) and DNase I (Sigma) before preparing single-cell suspensions for staining. FDCs were isolated as described previously^35^. Briefly, spleens were digested in 1 ml dissociation media (complete RPMI, 1 mg ml^−1^ collagenase D, 200 μg ml^−1^ dispase and 100 μg ml^−1^ DNase I) for 30 min in a 37 °C, 5% CO_2_ incubator. Cell suspensions were then gently triturated through P1000 pipette tips and incubated for a further 30 min. Dissociated cells were washed with complete RPMI to remove excess digestive enzymes. To stain for surface markers, cells were incubated with each antibody layer for 30 min in the dark at 4 °C and washed thoroughly with ice-cold FACS buffer (2% FCS and 0.1% NaN_3_ in PBS) between each layer. Intracellular staining was performed using the Intracellular Staining Kit (eBioscience) according to the manufacturer’s instructions. Serum IL-6 was measured using CBA bead assays (BD Biosciences) following the manufacturer’s instructions. A BD LSRII Flow Cytometer with FACSDiva software were used for flow cytometry acquisition and FlowJo (Tree Star) was used for analysis. FACS Aria (BD Biosciences) was used for cell sorting, cells were collected in FCS. Tonsillar T cells were sorted using the following strategy: CD4^+^ CD25^−^ CD44^low^ CXCR5^int/low^ PD-1^int/low^ naïve T cells, CD4^+^ CD25^−^ CD44^high^ CXCR5^int/low^ PD-1^int/low^ non-Tfh effector cells, CD4^+^ CD25^+^ CD44^int^ CXCR5^int/low^ PD-1^int/low^ Tregs, CD4^+^ CD25^−^ CXCR5^high^ PD-1^high^ Tfh cells, CD4^+^ CD25^+^ CXCR5^high^ PD-1^high^ Tfr cells.

### Luciferase reporter assay

HEK293T cells cultured in DMEM media (Gibco) supplemented with 10% heat-inactivated FCS, 100 U penicillin–streptomycin (Gibco) and 2 mM L-Glutamine (Gibco) in 24-well plates were co-transfected with pmirGLO (Promega) luciferase reporter plasmids containing either wild-type or mutated putative miR-146a minimal target sequences (Supplementary Table 1; designed according to pmirGLO’s protocol) and *mir*Vana miR-146a mimic (Life Technologies) or *mir*Vana negative control #1 (scramble; Life Technologies) using Lipofectamine 2000 (Life Technologies) according to the manufacturer’s instructions. The luciferase construct containing *Stat1* 3′UTR was purchased from Genecopoeia. After 24 h, 3 × 10^5^ transfected cells were moved into opaque flat-bottom 96-well plates and incubated for another 24 h. Cells were then harvested and luciferase activity was assessed using Dual-Glo Luciferase Assay System (Promega) according to manufacturer’s protocol.

### Microarray

Total RNA extraction from sorted tonsillar cells was done using the RNeasy Kit (QIAGEN) according to the manufacturer’s protocol. RNA quantity and quality was determined using a Nanodrop 1000 and an Agilent 2100 Bioanalyzer, respectively. Hybridization on Agilent Human miRNA Microarray (V1) Kit was performed at The Ramaciotti Center for Genomics (University of New South Wales, Australia). Triplicates (for human miRNA array) were collected and averaged in GeneSpring. The microarray data has been deposited in NCBI's Gene Expression Omnibus and is accessible through GEO Series accession number GSE64833.

### Real-time PCR analysis

RNA was extracted using Trizol reagent (Life Technologies) according to the manufacturer’s protocol, and complementary DNA was synthesized using the miScript II reverse transcription Kit (QIAGEN) following the manufacturer’s instructions. Quantitative PCR to detect for miR-146a expression was performed using the miScript SYBR Green PCR Kit (QIAGEN) and miScript Primer Assays (QIAGEN) to amplify mature *Mir146a* and the U6 small nuclear RNA. *Il6* and β-actin mRNAs were amplified using specific primer pairs purchased from Origene Technologies(*Il6*F: 3′- TACCACTTCACAAGTCGGAGGC -5′; *Il6*R: 3′- CTGCAAGTGCATCATCGTTGTTC -5′; *Actin*F: 3′- CATTGCTGACAGGATGCAGAAGG -5′; *Actin*R:3′- TGCTGGAAGGTGGACAGTGAGG -5′). Quantitative PCR was performed on an Applied Biosystems 7900 machine. The expression of *Mir146a* was normalized to U6, while the expression of *Il6* was normalized to β-actin. The gene specific expression change was calculated using the 2^−ΔΔCt^ method.

### Statistical analysis

Statistical significance was determined using the non-parametric Mann–Whitney *U*-test unless specified otherwise. The sample size was chosen so that it would be possible to conduct Mann–Whitney test with at least 95% confidence. Consequently in most experiments, at least five mice per group were used. All statistical analyses were performed using the GraphPad Prism software. NS, not significant, *P<0.05, **P<0.01, ***P<0.001, ****P<0.0001.

## Author contributions

A.P. designed and performed experiments, analyzed the data and wrote the manuscript. M.S. performed the human miRNA array and miR-146a-deficient mouse microarray experiments and analyzed the data. N.J.W., I.P., S.K.L. performed experiments. X.T.D. and M.A.J. performed experiments on *Slamf1* transgenic mice, analyzed the data and reviewed the manuscript. A.H. contributed the anti-ICOSL antibody and reviewed the manuscript. J.L.Z. reviewed the manuscript. R.C. contributed data on the transcription factor analysis and reviewed the manuscript. V.A. performed experiments and reviewed the manuscript. C.G.V. designed experiments, wrote the manuscript and supervised the study.

## Additional information

**How to cite this article:** Pratama, A. *et al*. MicroRNA-146a regulates ICOS–ICOSL signalling to limit accumulation of T follicular helper cells and germinal centers. *Nat. Commun.* 6:6436 doi: 10.1038/ncomms7436 (2015).

## Supplementary Material

Supplementary Information Supplementary Figures 1-15 and Supplementary Table 1
