# Supplementary material for: MicroRNA-146a regulates ICOS–ICOSL signalling to limit accumulation of T follicular helper cells and germinal centres
Source: Nat Commun. 2015 Mar 6;6:6436. doi: 10.1038/ncomms7436 (PMC4366510; doi:10.1038/ncomms7436)
Supplement: Supplementary Information — Supplementary Figures 1-15 and Supplementary Table 1 [file ncomms7436-s1.pdf]

Supplementary Figure 1

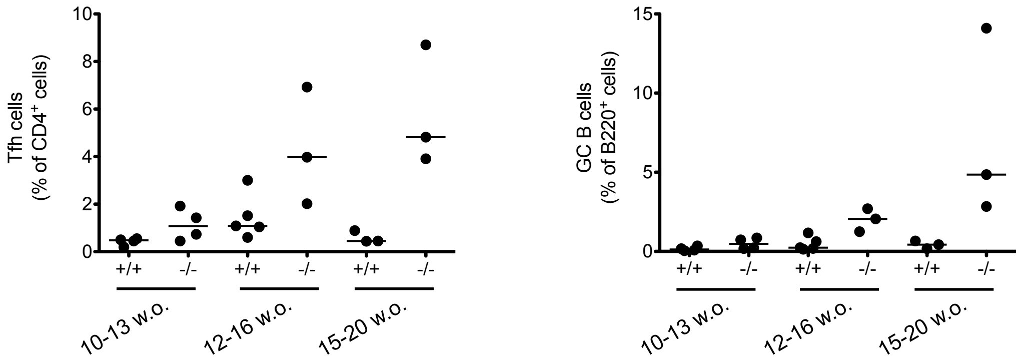

Supplementary Figure 1 Age-dependent accumulation of Tfh and GC B cells. Dot plots showing the percentage of Tfh (left) and GC B (right) cells in mice lacking miR-146a (-/-) or wild-type littermates (+/+) of different age groups. w.o. = weeks old. Each symbol represents one mouse and the horizontal bars represent the median values.

Supplementary Figure 2

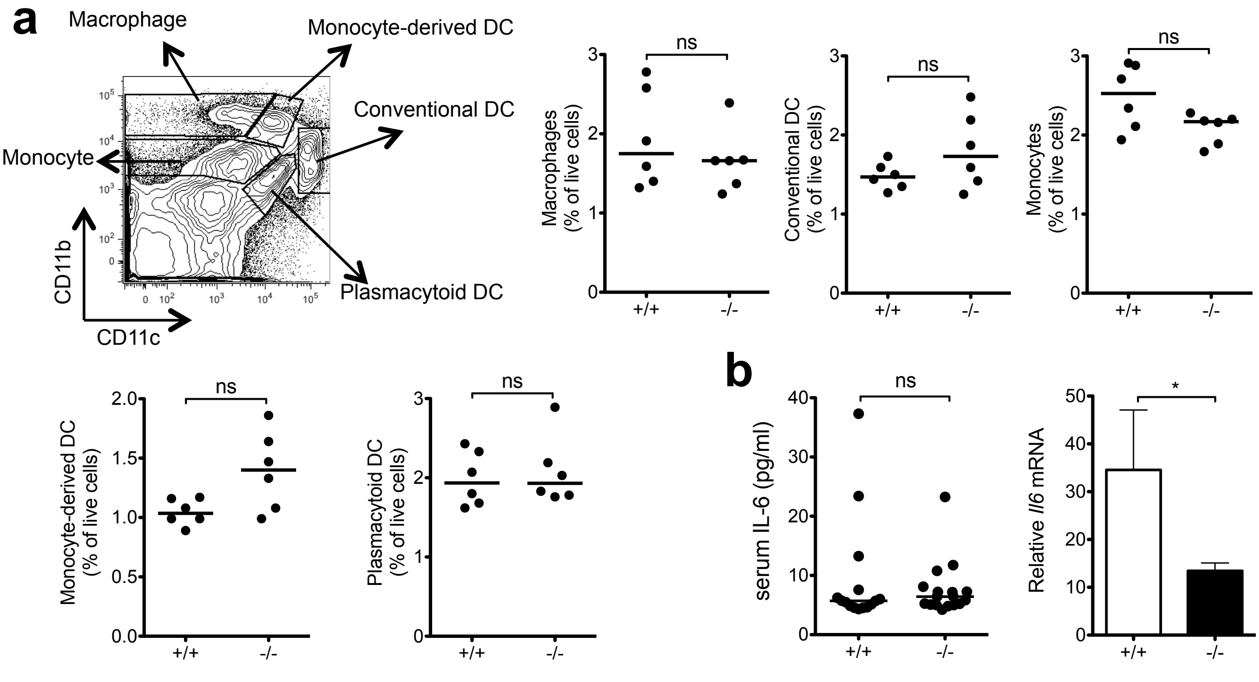

Supplementary Figure 2 No excessive myeloid proliferation or secretion of IL-6 in 12 to 16-week old miR146a-deficient mice. (**a**) Gating strategy and percentages of myeloid cell subsets in unimmunized 12 to 16-week old *Mir146a*$^{+/+}$ and *Mir146a*$^{-/-}$ mice. Data are representative of three independent experiments. (**b**) Serum concentration of IL-6 in unimmunized *Mir146a*$^{+/+}$ and *Mir146a*$^{-/-}$ mice (left) and relative amount of *Il6* mRNA (normalized to β-actin) in unstimulated *Mir146a*$^{+/+}$ and *Mir146a*$^{-/-}$ CD11c^{high} splenic dendritic cells (right) at 12-16 weeks of age. Each symbol represents one mouse and the horizontal bars represent the median values. Height of bar graph in **b** represents mean and error bar represents s.d. Statistical significance was determined using a Mann-Whitney *U* test (**a, b** left) or unpaired Student's *t* test (**b**, right). ns = not significant, * $P < 0.05$.

Supplementary Figure 3

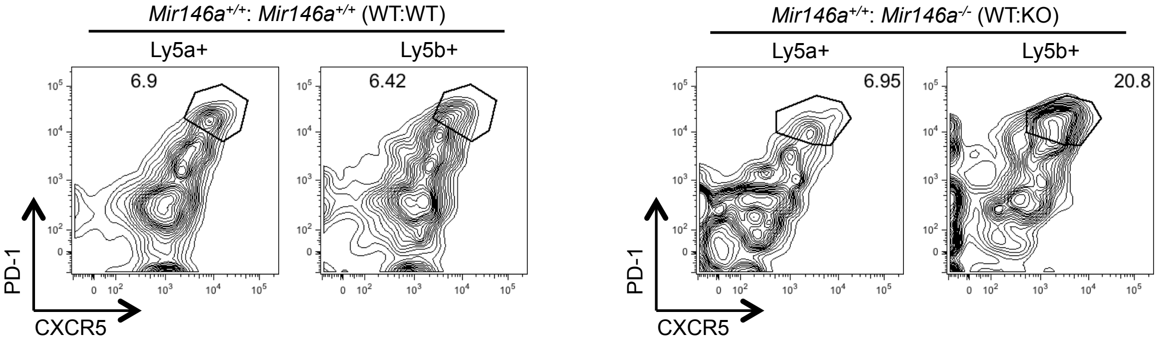

Supplementary Figure 3 Cell-autonomous accumulation of miR-146a-deficient Tfh cells in *Mir146a*$^{+/+}$: *Mir146a*$^{-/-}$ mixed bone marrow chimeric mice. Flow cytometric contour plots showing the percentages of Tfh cells from Ly5a^{+}.*Mir146a*$^{+/+}$: Ly5b^{+}.*Mir146a*$^{-/-}$ mixed bone marrow chimeras and control Ly5a^{+}.*Mir146a*$^{+/+}$: Ly5b^{+}.*Mir146a*$^{+/+}$ chimeras analyzed 14 weeks post-reconstitution. Mice were immunized with sheep red blood cells and sacrificed 7 days later. Analysis of WT:WT and WT:KO chimeras were performed on different days.

Supplementary Figure 4

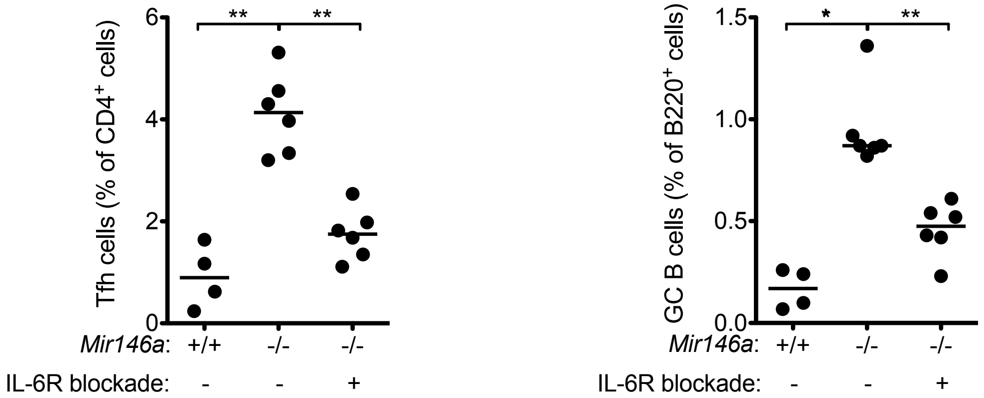

Supplementary Figure 4 The effect of complete IL-6R blockade in Tfh and GC B cell accumulation in the absence of miR-146a. Percentages of Tfh (left) and GC B (right) cells in wild-type (+/+) and miR-146a-deficient mice (-/-). For the IL-6R blockade, animals were treated with 150 µg anti-IL-6R blocking antibody i.p. every 5 days for 8 weeks since they were 8 weeks old. Each symbol represents one mouse and the horizontal bars represent the median values. Data are representative of two independent experiments. Statistical significance was determined using Mann-Whitney *U* test. * $P < 0.05$, ** $P < 0.01$.

Supplementary Figure 5

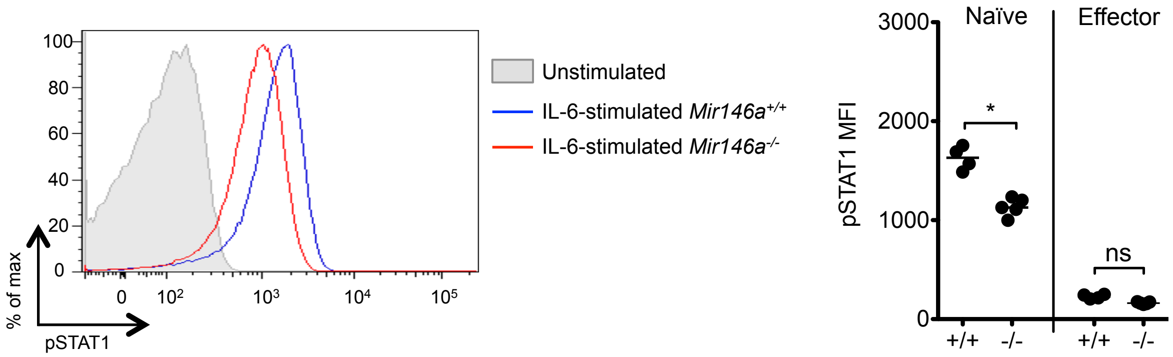

Supplementary Figure 5 Analysis of IL-6-induced STAT1 phosphorylation in miR-146a-deficient and wild-type T cells. Histograms (left) and dot plots (right) showing the amounts of STAT1 phosphorylation in naïve and effector $CD4^+$ T cells from miR-146a-sufficient (+/+) or -deficient (-/-) mice following acute stimulation with recombinant mouse IL-6 (20 ng/ml). Each symbol represents one mouse and the horizontal bars represent the median values. Data are representative of two independent experiments. Statistical significance was determined using Mann-Whitney *U* test. ns = not significant, * $P < 0.05$.

Supplementary Figure 6

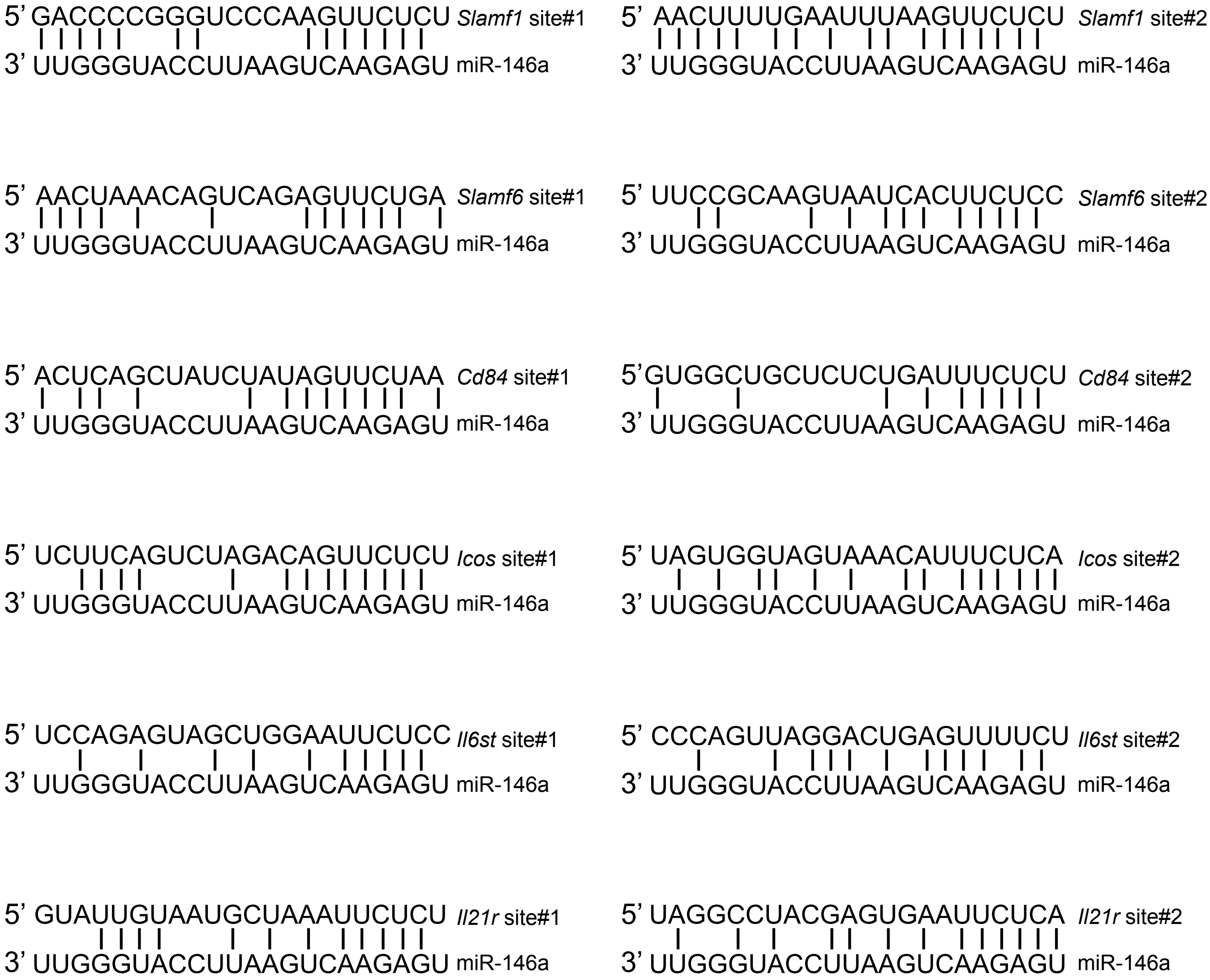

Supplementary Figure 6 Alignments of selected putative miR-146a targets. The minimal RNA sequences of *Slamf1, Slamf6, Cd84, Icos, Il6st*, and *Il21r* 3' untranslated regions (UTRs) that are predicted to bind to miR-146a are shown 5' to 3', whereas mature miR-146a sequence is shown from 3' to 5'. Vertical lines indicate possible base pairing.

Supplementary Figure 7

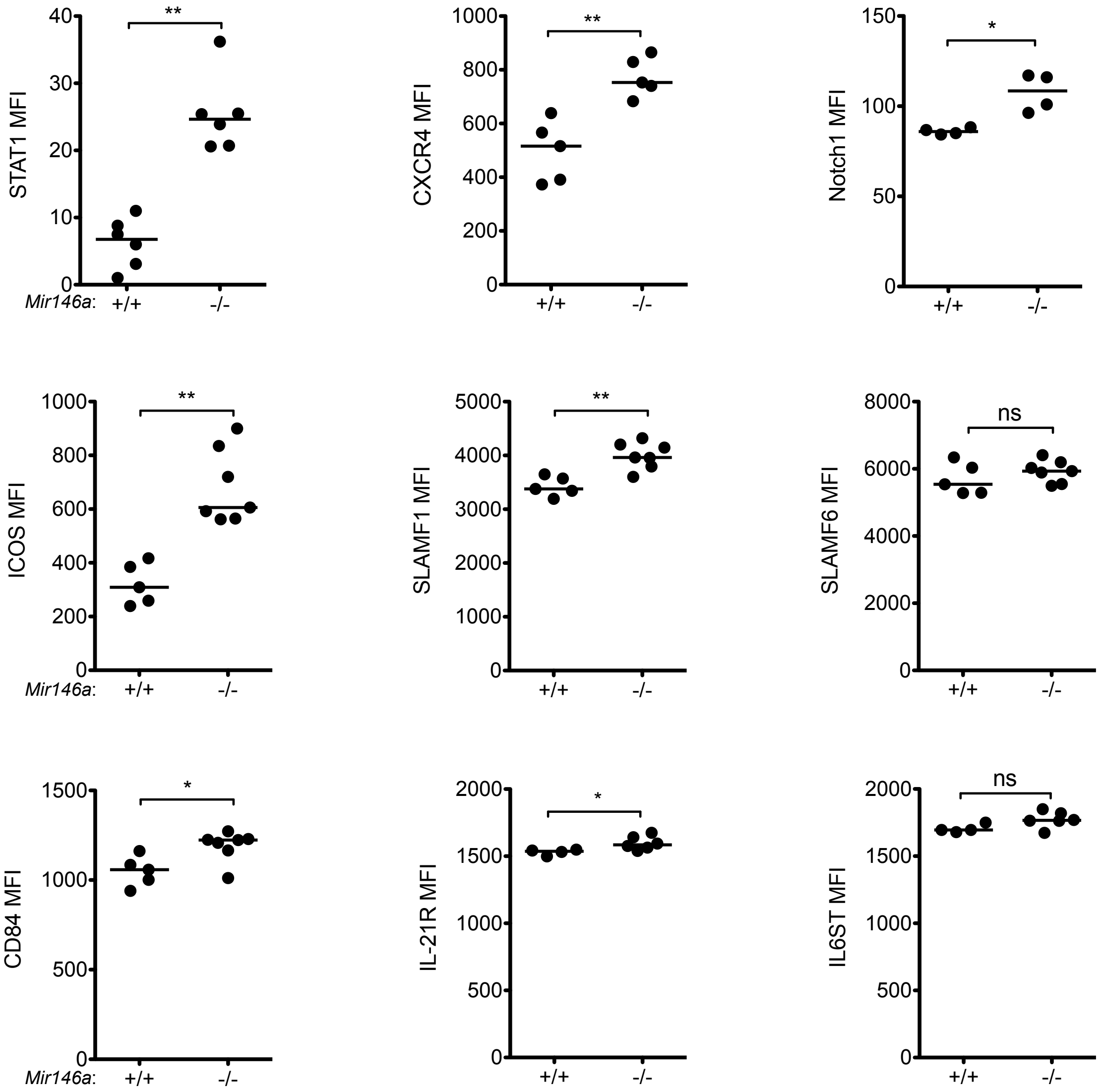

Supplementary Figure 7 Increased expression of the putative miR-146a targets in *Mir146a*$^{-/-}$ Tregs. Dot plots showing the geometric mean fluorescence intensity (MFI) of STAT1, CXCR4, Notch1, ICOS, SLAMF1, SLAMF6, CD84, IL-21R, and IL6ST on Tregs ($CD4^+$ $Foxp3^+$) from mice lacking miR-146a (-/-) or wild-type littermates (+/+). Data are representative of at least two independent experiments. Each symbol represents one mouse and the horizontal bars represent the median values. Statistical significance was determined using a Mann-Whitney *U* test. * $P < 0.05$, ** $P < 0.01$, ns = not significant.

Supplementary Figure 8

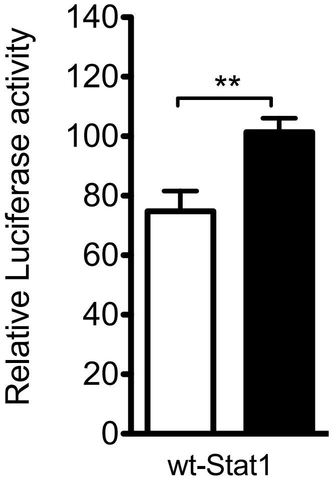

Supplementary Figure 8 Effects of miR-146a (open bar) and scramble (negative control) RNA (filled bar) expression on a luciferase reporter construct containing *Stat1* 3'UTR as relative luciferase activity (normalized to the *Renilla* control). Relative luciferase activity of cells transfected with miR-146a is expressed as a percentage of that transfected with the scramble RNA. Data are representative of at least two independent experiments. The heights of the bars represent the mean, and the error bars represent the s.d. of six technical replicates. Statistical significance was determined using unpaired Student's *t* test. ** $P < 0.01$.

Supplementary Figure 9

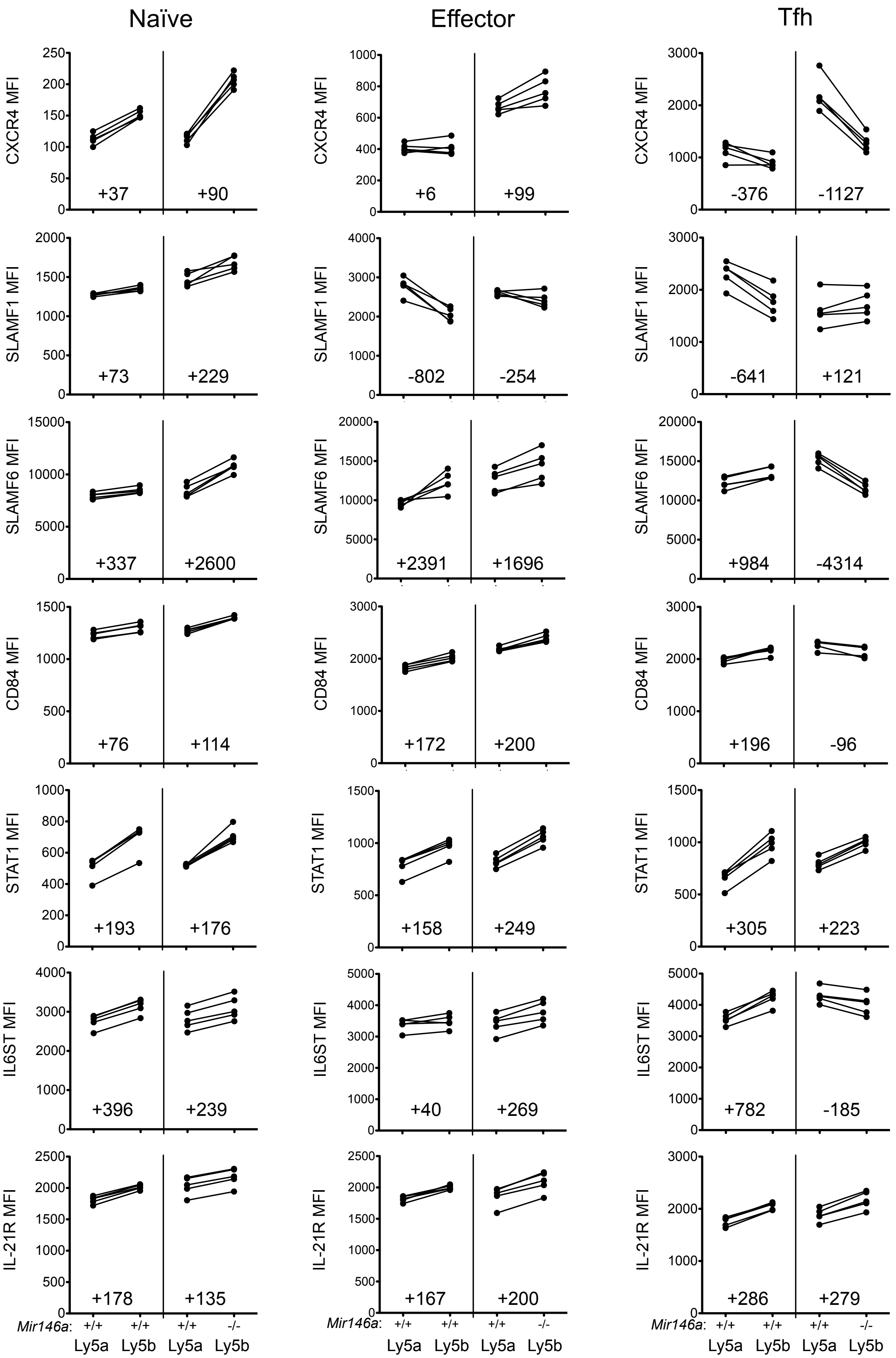

Supplementary Figure 9 Expression of putative miR-146a targets in *Mir146a*$^{-/-}$ T cells from mixed bone marrow chimeric mice. Dot plots showing the geometric mean fluorescence intensity (MFI) of CXCR4, SLAMF1, SLAMF6, CD84, STAT1, IL6ST and IL-21R on naive (CD4$^+$ CD44^{low}), effector (CD4$^+$ CD44^{high}), and Tfh (CXCR5^{high} PD-1^{high} Foxp3$^-$) cells from Ly5a$^+$.*Mir146a*$^{+/+}$: Ly5b$^+$.*Mir146a*$^{-/-}$ mixed bone marrow chimeras and control Ly5a$^+$.*Mir146a*$^{+/+}$: Ly5b$^+$.*Mir146a*$^{+/+}$ chimeras analyzed 16 weeks post-reconstitution. Data are representative of two independent experiments. Each symbol represents one mouse and the numbers below the symbols indicate the difference between the median MFI value of Ly5b$^+$ cells and that of Ly5a$^+$ cells in each chimera group. Connecting lines between MFI values in Ly5a$^+$ and Ly5b$^+$ cells indicate that they are from the same chimeric mouse.

Supplementary Figure 10

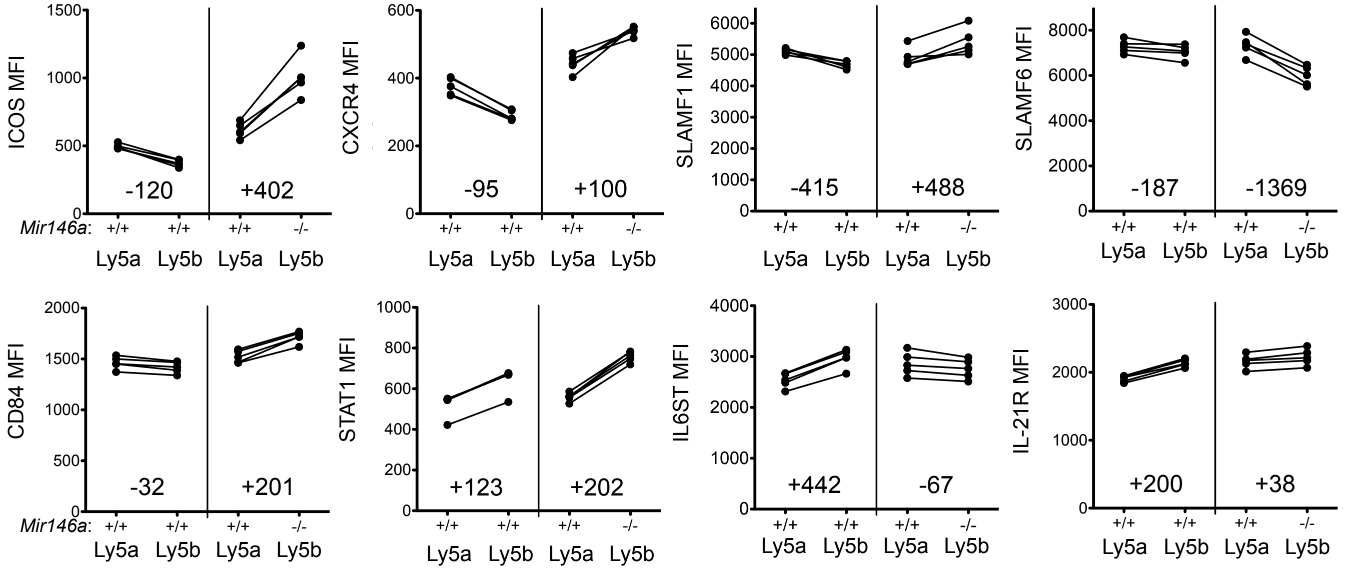

Supplementary Figure 10 Expression of putative miR-146a targets in *Mir146a*$^{-/-}$ Tregs from mixed bone marrow chimeric mice. Dot plots showing the geometric mean fluorescence intensity (MFI) of ICOS, CXCR4, SLAMF1, SLAMF6, CD84, STAT1, IL6ST and IL-21R on Tregs (CD4$^+$ Foxp3$^+$) from Ly5a$^+$.*Mir146a*$^{+/+}$: Ly5b$^+$.*Mir146a*$^{-/-}$ mixed bone marrow chimeras and control Ly5a$^+$.*Mir146a*$^{+/+}$: Ly5b$^+$.*Mir146a*$^{+/+}$ chimeras analyzed 16 weeks post-reconstitution. Data are representative of two independent experiments. Each symbol represents one mouse and the numbers below the symbols indicate the difference between the median MFI value of Ly5b$^+$ cells and that of Ly5a$^+$ cells in each chimera group. Connecting lines between MFI values in Ly5a$^+$ and Ly5b$^+$ cells indicate that they are from the same chimeric mouse.

Supplementary Figure 11

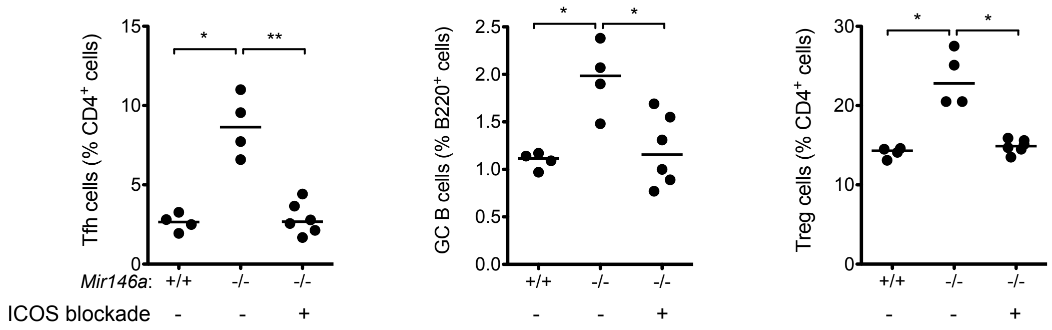

Supplementary Figure 11 Partial ICOS signaling blockade corrected the Tfh, GC B and Treg cell accumulation in miR-146a-deficient mice. Dot plots showing the percentage of Tfh (left), GC B (middle), and Treg (right) cells in 9-week old control wild-type (+/+) and miR-146a-deficient mice (-/-) treated with 25 μg anti-ICOSL blocking antibody or PBS control on day 0, 3, and 6 following SRBC immunization. Mice were taken down on day 7 post-immunization. Each symbol represents one mouse and the horizontal bars represent the median values. Statistical significance was determined using a Mann-Whitney *U* test. * $P < 0.05$, ** $P < 0.01$.

Supplementary Figure 12

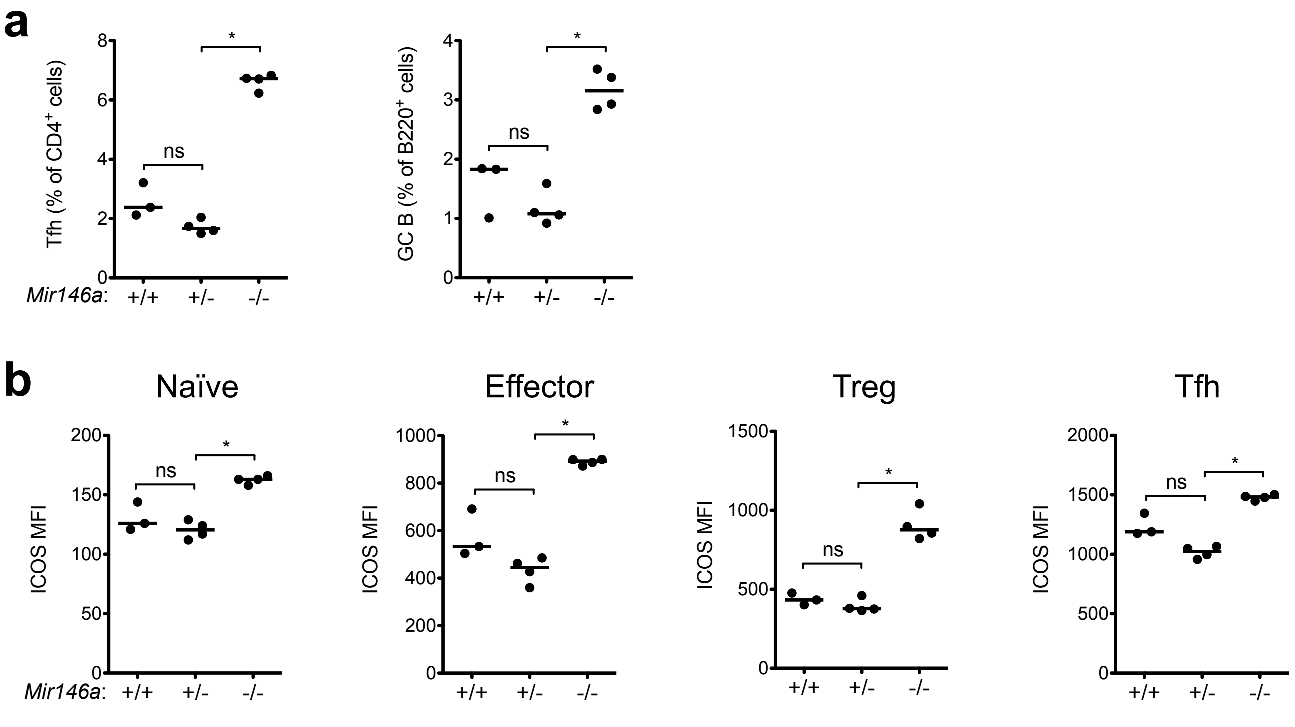

Supplementary Figure 12 No accumulation of Tfh or GC B cells or increased ICOS expression in *Mir146a*$^{+/-}$ mice. Dot plots showing the percentage of Tfh (**a**, left) and GC B (**a**, right) cells and the geometric MFI of ICOS on naïve, effector, Treg and Tfh cells (**b**) from 16-week old *Mir146a*$^{+/+}$, *Mir146a*$^{+/-}$, and *Mir146a*$^{-/-}$ mice. Each symbol represents one mouse and the horizontal bars represent the median values. Data are representative of two independent experiments. Statistical significance was determined using a Mann-Whitney *U* test. ns = not significant, * $P < 0.05$.

Supplementary Figure 13

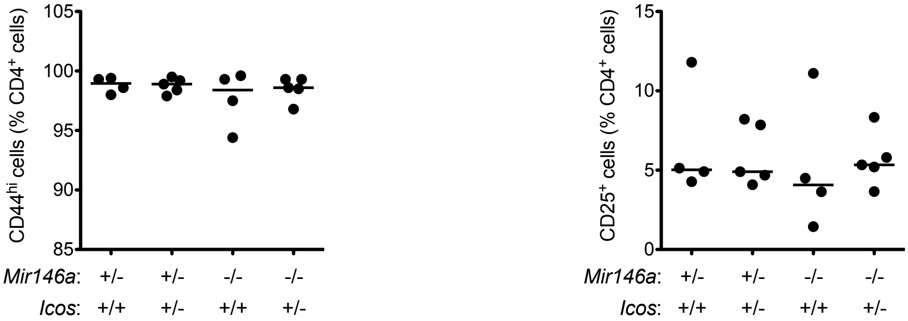

Supplementary Figure 13 Similar activation status of miR-146a-sufficient and -deficient T cells following adoptive transfer into Rag1-deficient mice. Dot plots showing the percentage of CD44^{high} (left) and CD25^{+} (right) cells amongst CD4^{+} T cells from either *Mir146a*$^{+/-}$ *Icos*$^{+/+}$, *Mir146a*$^{+/-}$ *Icos*$^{+/-}$, *Mir146a*$^{-/-}$ *Icos*$^{+/+}$, or *Mir146a*$^{-/-}$ *Icos*$^{+/-}$ mice at day 14 after adoptive transfer into *Rag1*$^{-/-}$ recipients but prior to SRBC immunization. The adoptive transfer strategy is shown in **Fig. 6e**. Each symbol represents one mouse and the horizontal bars represent the median values. No statistically significant difference was observed between any groups.

Supplementary Figure 14

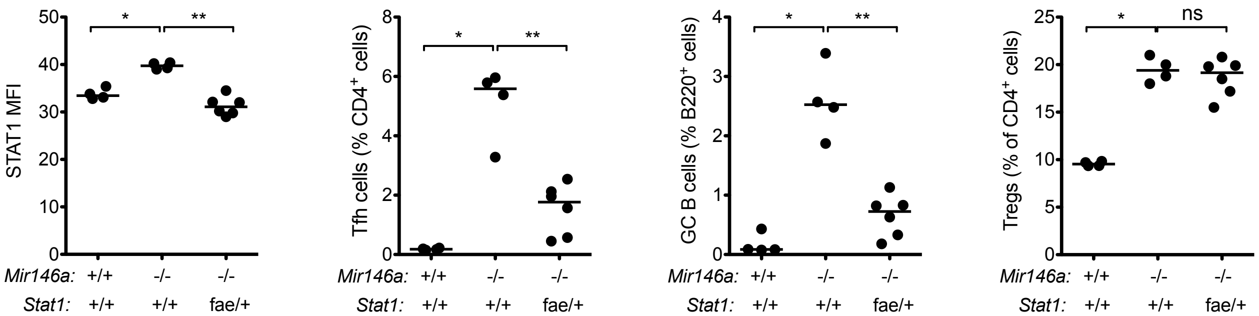

Supplementary Figure 14 MiR-146a-driven Tfh cell accumulation was partially corrected in the presence of *Stat1* partial gene deficiency. Dot plots showing the geometric mean fluorescence intensity (MFI) of STAT1 in Tregs and the percentage of Tfh, GC B, and Treg cells in *Mir146a*$^{+/+}$ *Stat1*$^{+/+}$, *Mir146a*$^{-/-}$ *Stat1*$^{+/+}$, and *Mir146a*$^{-/-}$ *Stat1*$^{fae/+}$ mice. Each symbol represents one mouse and the horizontal bars represent the median values. Statistical significance was determined using a Mann-Whitney *U* test. ns = not significant, * $P < 0.05$, ** $P < 0.01$.

Supplementary Figure 15

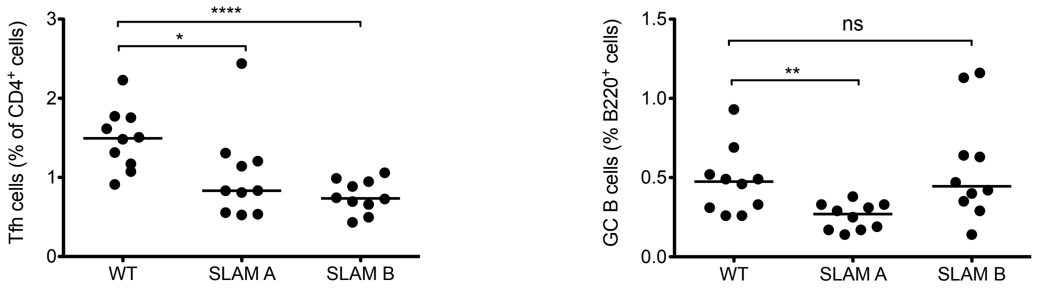

Supplementary Figure 15 Overexpression of SLAMF1 is not sufficient to cause spontaneous Tfh cell accumulation. Dot plots showing the percentage of Tfh (left) and GC B (right) cells in NOD.*Nkrp1b*.Tg (*Slamf1*)1 (SLAM A), NOD.*Nkrp1b*.Tg (*Slamf1*)2 (SLAM B) mice, and control wild-type littermates analyzed at 16 weeks of age. Each symbol represents one mouse and the horizontal bars represent the median values. Statistical significance was determined using a Mann-Whitney *U* test. * $P < 0.05$, ** $P < 0.01$, **** $P < 0.0001$.

Supplementary Table 1 Putative miR-146a minimal target regions used in the luciferase reporter assay.

Target name	Sequence (sense strand)	Sequence (antisense strand)
Wt-Icos (1 site)	aaac tagcggccgc tagt ttcttcagtctagacagttctcttct	ctaga gaagagaactgtctagactgaagaa acta gcggccgcta gttt
Mut-Icos (1 site)	aaac tagcggccgc tagt ttcttcagtctagacaaaaaaattct	ctaga gaatttttttgtctagactgaagaa acta gcggccgcta gttt
Wt-Icos (2 sites)	aaac tagcggccgc tagt ttcttcagtctagacagttctc ttct tagtggtagtaaacatttctca gt	ctaga ctgagaaatgtttactaccactaagaagagaactgtctagactgaagaa acta gcggccgcta gttt
Mut-Icos (2 sites)	aaac tagcggccgc tagt ttcttcagtctagacagaagac ttct tagtccaagtaaacatttgaga gt	ctaga ctctcaaatgtttacttggactaagaagtcttctgtctagactgaagaa acta gcggccgcta gttt
Wt-Slamf1	aaac tagcggccgc tagt ggaccccgggtcccaagttctc tgtgac gaacttttgaatttaagttctc tgttt	ctaga aacagagaacttaaattcaaaagttcgtcacagagaacttgggacccggg gtcc acta gcggccgcta gttt
Mut-Slamf1	aaac tagcggccgc tagt ggaccccgggtcccaaaaaaaa tgtgac gaacttttgaatttaaaaaaaa tgttt	ctaga aacattttttttaaattcaaaagttcgtcacattttttttgggacccggggtcc acta gcggccgcta gttt
Wt-Slamf6	aaac tagcggccgc tagt caactaaacagtcagagttctg atggac agattgattccgcaagtaatcacttctc cttct	ctaga gaaggagaagtgattacttgcggaatcaatctgtccatcagaactctgactg tttagttg acta gcggccgcta gttt
Mut-Slamf6	aaac tagcggccgc tagt caactaaacagtcagaaaaaaa atggac agattgattccgcaagtaatcaaaaaaa cttct	ctaga gaagtttttttgattacttgcggaatcaatctgtccattttttttctgactgtttagttg acta gcggccgcta gttt
Wt-Cd84	aaac tagcggccgc tagt aactcagctatctatagttcta agtatc agtggctgctctctgatttctc ttctt	ctaga agaagagaaatcagagagcagccactgatacttagaactatagatagctg agtt acta gcggccgcta gttt
Mut-Cd84	aaac tagcggccgc tagt aactcagctatctataaaaaaa agtatc agtggctgctctctgaaaaaaa ttctt	ctaga agaatttttttcagagagcagccactgatacttttttttatagatagctgagtt acta gcggccgcta gttt
Wt-Il21r	aaac tagcggccgc tagt tgtattgtaatgctaaattctctacccaa agttctaggcctacgagtgaattctcatgttt	ctaga aacatgagaattcactcgtaggcctagaactttgggtagagaatttagcatta caataca acta gcggccgcta gttt
Mut-Il21r	aaac tagcggccgc tagt tgtattgtaatgctaaaaaaaatacccaa aaaaaaaggcctacgagtgaaaaaaaaatgttt	ctaga aacattttttttcactcgtaggcctttttttttgggtattttttttagcattacaataca acta gcggccgcta gttt
Wt-Il6st	aaac tagcggccgc tagt tccagagtagctggaattctc cttcaa cccagttaggactgagttttc tatgt	ctaga catagaaaactcagtcctaactgggttgaaggagaattccagctactctgg a acta gcggccgcta gttt
Mut-Il6st	aaac tagcggccgc tagt tccagagtagctggaaaaaaa cttcaa cccagttaggactgaaaaaaa tatgt	ctaga catatttttttcagtcctaactgggttgaagtttttttccagctactctgga acta gcggccgcta gttt
